# The effect of clinical interventions on hospital readmissions: a meta-review of published meta-analyses

**DOI:** 10.1186/2045-4015-2-1

**Published:** 2013-01-23

**Authors:** Jochanan Benbassat, Mark I Taragin

**Affiliations:** 1 JDC Brookdale Institute, Health Policy Research Program, PO Box 3886, Jerusalem, 91037, Israel; 2 National Insurance Institute, Jerusalem, Israel

**Keywords:** Patient readmissions, Clinical trials, Quality assurance of care, Continuity of care, Disease management, Home care

## Abstract

**Background:**

The economic impact and ease of measurement of all-cause hospital readmission rates (HRR) have led to the current debate as to whether they are reducible, and whether they should be used as a publicly reported quality indicators of medical care.

**Objective:**

To assess the efficacy of broad clinical interventions in preventing HRR of patients with chronic diseases

**Method:**

A meta-review of published systematic reviews of randomized controlled trials (RCTs) of clinical interventions that have included HRR among the patients' outcomes of interest.

**Main findings:**

Meta-analyses of RCTs have consistently found that, *in the community,* disease management programs significantly reduced HRR in patients with heart failure, coronary heart disease and bronchial asthma, but not in patients with stroke and in unselected patients with chronic disorders. *Inhospital* interventions, such as discharge planning, pharmacological consultations and multidisciplinary care, and *community* interventions in patients with chronic obstructive pulmonary diseases had an inconsistent effect on HRR.

**Main study limitation:**

Despite their economic impact and ease of measurement, HRR are not the most important outcome of patient care, and efforts aimed at their reduction may compromise patients' health by reducing also justified re-admissions.

**Conclusions:**

The efficacy of *inhospital* interventions in reducing HRR is in need of further study. In patients with heart diseases and bronchial asthma, HRR may be considered as a publicly reported quality indicator of *community care*, provided that future research confirms that efforts to reduce HRR do not adversely affect other patients’ outcomes, such as mortality, functional capacity and quality of life. Future research should also focus on the reasons for the higher efficacy of community interventions in patients with heart diseases and bronchial asthma than in those with other chronic diseases.

## Introduction

In the US, the proportion of hospital readmissions within 30 days after discharge has been stable over the last decade, and has fluctuated around 18% for patients with pneumonia, 20% for myocardial infarction and 24% for heart failure [1]. Some readmissions, such as those due to the natural history of the disease, unrelated medical conditions or non-health-related causes, are probably unavoidable. However, the variability in hospital readmission rates (HRR) by discharge destinations [2] and across hospitals [3,4] suggests that some readmissions are due to modifiable causes, such as sub-optimal medical care before or after discharge.

Examples of sub-optimal care *before* hospital discharge are failure to resolve the patient's problem [5] and to provide discharge letters [6]; unstable doses of therapy, fever and intravenous fluids upon discharge [7]; wrong medications, and unaddressed test results [8]. Examples of sub-optimal care *after* hospital discharge are failure to provide patients with a smooth transition to the community and appropriate follow-up [9,10]. Therefore, attempts to improve patients' outcomes have consisted of improving the quality of inhospital and outpatient care [11], and its continuity during patient transfers between different settings [12].

Indeed, there is evidence that many interventions improve important patient outcomes, such as mortality, activities of daily living, quality of life and satisfaction with care [13,14], and, therefore, should be implemented even if they do not reduce HRR. Still, HRR have drawn interest mainly because of their economic impact, their ease of measurement, and because of the ongoing debate whether they should be used as a publicly reported quality indicator of hospital care [1]. Furthermore, HRR may be viewed as an, albeit imperfect, proxy of poor health or healthcare in patients with chronic disorders. Therefore, HRR have been the subject of observational and experimental studies, which, in turn, have been subject to systematic reviews.

When there are multiple reviews on an important topic, *meta-reviews* of individual systematic reviews may help evidence-based decision-making [15]. Individual systematic reviews may differ in focus (i.e., profession of care-provider, type of intervention, patient population) and in time periods covered by their literature searches. Still, comparisons among reviews may either confirm the consistency of their conclusions, or provide important insights into the causes for their conflicting interpretations.

We know of only three meta-reviews of individual reviews of the effect of interventions on HRR. The first one was restricted to the hospital setting [13], the second – to patients with heart failure [16], and the third – to integrated care programs for chronically ill patients [17]. The present study is an updated meta-review of published systematic reviews of the effect of broad clinical interventions on HRR. The term "broad clinical interventions", as used here, refers to basic, standard and all-purpose management modalities, as opposed to specific diagnostic (e.g., angiography) or treatment (e.g., laparoscopic surgery) interventions. An attempt is made to answer the questions: (a) which types of interventions are efficacious in reducing HRR? (b) In which settings? and (c) Which participants benefit most?

## Methods

### Study design

The present meta-review is restricted to published systematic reviews of randomized controlled trials (RCTs) that compare patients who received conventional care with those who had one or more of the following broad clinical interventions [13,18].

Hospital – based interventions

1. *Discharge planning:* an all-inclusive term for providing patients with information about their disease, or / and educating patients for following prescribed treatment plans, or / and ensuring communication between the members of the medical team, or / and assessing the patient's support networks, or / and post-discharge services, or / and arranging for follow-up.

2. *Pharmacological consultations:* a review of the patient's medications by a pharmacist with a view of improving the patient's knowledge of, and compliance with, the treatment regimen, identifying medication \ discrepancies, drug reactions or interactions.

3. *Geriatric consultations, or comprehensive geriatric assessment programs:* a review by geriatricians with advice on diagnostic evaluations, therapy, rehabilitation, social care and placement.

4. *Case management:* a systematic approach to care of patients with multiple chronic disorders.

5. *Disease management*: a systematic approach to care of patients with a specific chronic disease, such as stroke or congestive heart failure. Disease management programs may be implemented in specific in hospital units or through clinical guidelines / pathways.

6. *In hospital management units*: hospital wards, staffed by doctors, nurses and other health professionals for diagnostic assessment, therapy, rehabilitation and placement of patients in order to intensify post discharge care, identify effective community services and enhance primary care access.

Community – based interventions

1. *Periodic home visits by professional care providers*, single or multi-disciplinary. The service may be provided either by a *"Disease manager" (*for patients with a specific chronic disease), or by a *"Case manager"* (for patients with multiple diseases).

2. *Self-management:* Patient education for self-monitoring with a view of enabling patients to assume responsibility for managing one or more aspects of their disease by medication dosage adjustment or by recognizing a need for medical assistance.

3. *Telephone follow-up* aimed at exchanging information, providing health education and advice, managing symptoms, recognizing complications and giving reassurance.

4. *Telemonitoring* of physiological variables measured by patients at home.

5. *Community - based rehabilitation programs*

6. *Day care*

7. *Hospital at home*

The participants in the RCTs were inpatients or outpatients who were believed to be at risk of increased HRR. It should be noted that most systematic reviews of the effect of such intervention synthesize a heterogeneous collection of primary studies that may differ in duration of follow-up, frequency of contacts with care providers and their professional backgrounds.

We used all-cause HRR as the outcome of interest, and, unless otherwise stated, ignored reported rates of disease specific readmissions, or of readmissions that were believed by the authors to be preventable. We are aware that disease specific readmission rates are a better indicator of the efficacy of interventions than all-cause HRR. Still, the vast majority of published systematic reviews address all-cause HRR, which are readily available from hospital databases. On the other hand, the distinction between preventable and unavoidable readmissions requires a painstaking review of medical records, and even then, the reliability (i.e., agreement between evaluators) of the distinction is only moderate [19,20].

### Electronic searches and selection of systematic reviews

We searched the literature without language restriction, using the electronic data bases and key terms listed in Additional file 1: Appendix 1 from inception until September 2012, as well as the reference lists of the retrieved articles. We did not seek further information from authors of individual systematic reviews and we did not review the gray literature. One of us (JB) screened the identified titles / abstracts and excluded studies that obviously did not meet the inclusion criteria, namely, systematic reviews of RCTs of the effect of clinical interventions on HRR that presented their findings either in terms of risk / odds ratios and 95% confidence intervals, or in other presentations formats. Both authors reviewed the full text of the remaining papers, and, after resolving differences in opinions by discussions, further excluded systematic reviews that did not meet the inclusion criteria or met / fulfilled one or more of the following exclusion criteria:

1. Duplicate systematic reviews or availability of an updated systematic review.

2. Studies of pediatric, obstetric, terminal and psychiatric patients.

3. Studies of the effect of disease specific diagnostic (e.g., angiography) or treatment (e.g., laparoscopic surgery) interventions on hospital readmissions.

4. Protocols of planned studies and models predicting readmissions, position statements and methodology papers.

5. Reviews that failed to identify any eligible studies in the literature search.

6. Interventions targeted at care providers rather than at patients.

7. Primary research studies, i.e., reports of single trials.

### Data extraction

We used a predetermined format to stratify the selected systematic reviews by method of data synthesis (meta-analyses, or systematic reviews that presented their findings using other formats), setting of intervention (hospital only, or community with and without inhospital interventions), patient populations (unselected patients, or patients with specific disorders) and type of intervention (e.g., discharge planning, home care). Some systematic reviews synthesized RCTs at both settings of care, involving two or more patient populations, or two or more types of interventions, and, therefore, they are referred to more than once in the same or different tables.

Most selected systematic reviews were meta-analyses that presented their findings in terms of risk or odds ratios with 95% confidence intervals. The advantage of meta-analyses is that they take into account the power of the primary studies. However, the heterogeneity in the implementation of the same clinical interventions, in the professionals who implemented them, in the patient populations and in the duration of follow-up detracts from the credibility of the synthesis of various RCTs. Some reviews did not discern between RCTs and non-randomized controlled trials. In such cases, we retrieved the primary studies, selected the RCTs only, and re-synthesized their results using the Meta-Analyst software [21].

The remaining systematic reviews synthesized their findings using formats other than risk ratios, mostly in terms of proportion of RCTs reporting significantly reduced HRR. Their advantage is that they present *separately* the results of the primary RCTs, and thereby avoid averaging the results of possibly heterogeneous studies. However, by implicitly assigning the same weight to the reviewed RCTs, such systematic reviews may overemphasize studies that failed to detect a significant reduction in HRR because of low power.

### Quality assessment of the systematic reviews

One of us (JB) assessed the quality of the identified reviews according to the 11 AMSTAR methodological criteria [22] on a 0–11 scale. We felt that these criteria were clear cut and that a single investigator was capable of applying them. Indeed, the AMSTAR scores in the present overview differed by 1 or less in 14 of the 15 meta-analyses that were included in the overview by Savard et al. [17].

We interpreted the 4^th^ AMSTAR criterion (use of status of publication as an inclusion criterion) as the presence of a reference to the grey literature or a statement that the authors of the reviewed RCTs were contacted for additional information. The 9^th^ AMSTAR criterion (use of appropriate methods to combine the study findings) was interpreted as requiring either an assessment of heterogeneity (in case of meta-analyses), or a presentation of the findings, which permitted a calculation of the proportion of RCTs that found significant differences in HRR (in case of other systematic reviews). The 'results' section is restricted to the findings of the meta-analyses only, regardless of their quality; the summary and conclusions in the 'discussion' section are based on meta-analyses with an AMSTAR score of 7 or more.

## Results

### Yield of the literature review

Our electronic searches identified a total of 1,668 titles / abstracts (Additional file 1: Appendix 1). Of these, it was clear from the abstract that 1,478 of them did not meet the selection criteria. After reviewing the full text of the remaining papers, we further excluded 97 systematic reviews (Additional file 2: Appendix 2). The searches of the reference sections of the remaining relevant studies revealed 6 additional systematic reviews, thereby bringing the number of studies that were included in the present meta-review to a total of 99. Of these, 57 were meta-analyses that presented their findings as *average* risk or odds ratios of HRR in intervention / control patients and their 95% confidence intervals (Additional file 3: Appendix 3), while the remaining 42 were systematic reviews that have synthesized their data using other methods (Additional file 4: Appendix 4).

### Included systematic reviews

Additional file 5: Appendix 5 and Additional file 6: Appendix 6 present the degree to which the selected meta-analyses and other systematic reviews, respectively, met the 11 AMSTAR methodological criteria [22]. The average quality of meta-analyses (8.0 +/− 1.9; range: 3–11) was higher than that of other systematic reviews (6.1 +/− 2.2; range: 2–11). The most common methodological limitations were failure to provide explicit statements that the literature was searched for reports regardless of their publication status, and failure to consider the quality of the reviewed studies in formulating the conclusions. The present meta-review is restricted to the findings of the 57 meta-analyses (Tables 1 and 2). A summary of the findings of the remaining 42 systematic reviews that synthesized their data using other methods is presented in Additional file 7: Appendix 7 and Additional file 8: Appendix 8.

**Table 1:** **Meta-analyses of randomized controlled trials of the effect of clinical interventions *before *hospital discharge on subsequent hospital readmission rates**

**Reference**	**Intervention**	**RCTs reporting HRR (n)**	**Total number of patients**	**RR or OR of HRR in intervention / control (95% CI)**
**(AMSTAR score)**				
**Disease management programs**				
Rotter et al. 2010 [23] (10)	Multidisciplinary care plans for patients with a specific clinical problem	6	672	0.60 (0.32-1.13)
Auer et al. 2008 [24] (7)	Multidimensional interventions for secondary prevention of acute coronary syndrome	3	405	0.78 (0.54-1.13)+
Kwan and Sandercock 2004[25] (10)	Care pathways for stroke	1	60	0.15 (0.04-0.59)
**Geriatric case management units**				
Ellis et al. 2011 [26] (7)	Comprehensive geriatric assessment	8	3,543	1.01 (0.87-1.17)
Van Craen et al. 2010 [27] (7)	Geriatric evaluation and management units	2	799	0.85 (0.65-1.11)
Baztán et al. 2009 [28] (8)	Acute geriatric units	3	*	1.11 (0.92-1.35)
Griffiths et al. 2007 [29] (9)	Nursing-led units for chronically ill or geriatric patients	3	493	0.63 (0.36-1.12)
Stuck et al. 1993 [30] (6)	Geriatric evaluation and management units	1	123	0.54 (0.26-1.11)+
**Geriatric consultations and assessment programs**				
Ellis et al. 2011 [26] (7)	Comprehensive geriatric assessment	1	279	1.25 (0.78-2.01)
Parker et al. 2002 [18] (9)	Comprehensive geriatric assessment	10	952	0.90 (0.73-1.11)
Stuck et al. 1993 [30] (6)	Geriatric consultation service	3+	449+	0.51 (0.27-0.95)+
**Pharmacological consultations**				
Kaboli et al. 2006 [31] (4)	Interventions by pharmacists: participation on rounds, medication reconciliation, drug-specific services	8	1,350	0.85 (0.49-1.46) +
**Discharge planning**				
Shepperd et al. 2010 [14] (9)	Discharge plan for inpatients. Studies that did not discern discharge planning from provision of care after discharge were excluded.	12	2,612	0.85 (0.74-0.97)
Parker et al. 2002 [18] (9)	Discharge arrangements for older patients : Comprehensive discharge protocols	6	1,958	0.93 (0.80-1.09)

HRR – Hospital readmission rates. RCT- Randomized controlled trial. RR – risk ratio. OR – odds ratio. CI – confidence intervals.

* not given + Datum not provided; calculated by the authors of the present meta-review.

**Table 2:** Meta-analyses of randomized controlled trials of the effect of clinical interventions before and/or after hospital discharge on hospital readmission rates

**Reference (AMSTAR score)**	**Intervention**	**RCTs reporting HRR (n)**	**Total number of patients**	**RR or OR of HRR in intervention / control (95% CI)**
**Heart failure**				
*Home care*				
Lambrinou et al. 2012 [32] (9)	Before hospital discharge: individualized patient education and discharge planning. After discharge: home care by cardiac community nurses or physicians	6	1,052	0.80 (0.70- 0.91)
Takeda et al. 2012 [33] (10)	Disease management with home visits and phone calls	7	2,199	0.75 (0.57-0.99)
	Multidisciplinary care	2	403	0.46 (0.30-0.69)
Tsai et al. 2005 [34] (6)	Chronic care model including: follow-up, planned visits, self-management (patient education)	16	4,324	0.73 (0.58-0.91)+
Whellan et al. 2005 [35] (3)	Discharge planning and disease management with home nursing	6	2,710	0.7 (0.6 - 0.9) +++
Roccaforte et al. 2005 [36] (9)	Disease management interventions before and after discharge (education, discharge planning, home or clinic care)	25	2,603	0.80 (0.68-0.94)
Holland et al. 2005 [37] (8)	Multidisciplinary interventions with 1–8 home visits	10	1,519	0.80 (0.71-0.89)
Kim and Soeken 2005 [38] (7)	Inhospital assessment and education with follow up by phone and home visits	4	817	0.75 (0.45-1.05)
Gonseth et al. 2004 [39] (9)	Discharge planning and patient education with home visits, or telephone follow-up, or clinic follow-up	16	4,440	0.88 (0.79-0.97)
Phillips et al. 2004 [40] (9)	Discharge planning with			
	A single home visit	3	476	0.76 (0.63-0.93)
	Home visits / frequent telephone contact	6	970	0.79 (0.69-0.91)
	Extended home care services	4	859	0.82 (0.68-1.00)
	Day Hospital services	1	234	0.25 (0.15-0.44)
McAlister et al. 2004 [41] (6)	Multidisciplinary team providing follow-up in a non-clinic setting	8	1,646	0.81 (0.72-0.91)
Gwadry et al. 2004 [42] (7)	Patient education and post discharge follow-up at home or by phone	8	1,239	0.79 (0.68-0.91)
*Self-management*				
Kozak et al. 2007 [43] (6)	Education for self-management before or after discharge			
	Face-to-face contact	9	1,747	0.42 (0.22-0.81) ++++
	Combined telephone and face to face contact	5	1,253	0.37 (0.21–0.64)++++
Jovicic et al. 2006 [44] (8)	Patients assume primary role in managing their health after receiving education before discharge, with limited follow-up phone calls or home visits after discharge	5	787	0.59 (0.44-0.80)
McAlister et al. 2004 [41] (6)	Enhanced patient self-care activities	4	568	0.73 (0.57-0.93)
*Pharmacist care*				
Koshman et al. 2008 [45] (8)	Pharmacist care in a multidisciplinary team, in hospital or in outpatient clinic, with or without home visits	11	2,026	0.71 (0.54-0.94)
*Telemonitoring / structured telephone support*				
Inglis et al. 2011 [46] (10)	Telemonitoring Structured telephone support	8	2,343	0.91 (0.84-0.99)
		11	4,295	0.92 (0.85-0.99)
Klersy et al. 2011 [47] (7)	Remote monitoring	18	5,312	0.87 (0.79-0.96)
*Exercise training*				
Lloyd-Williams et al. 2002 [48](5)	Exercise training	1	99	0.29 (0.11-0.84)
*Clinic follow up / telephone contact*				
Lambrinou et al. 2012 [32] (9)	Telephone follow-up	3	634	0.83 (0.66-1.04)
	Clinic follow-up	3	944	1.03 (0.75-1.40)
	Combination of settings	5	1,422	0.81 (0.64-1.03)
Takeda et al. 2012 [33] (10)	Clinic care	4	1,129	0.78 (0.48-1.26)
Kozak et al. 2007 [43] (6)	Education for self-management before or after discharge	7	1,671	0.67 (0.36-1.26) ++++
	Telephone contact			
Mistiaen and Poot 2006 [49] (8)	Telephone follow-up	2	258	0.67 (0.19-2.33)+
Phillips et al. 2005 [50] (8)	Specialist nurse-led clinics:			
	With hospital discharge planning	2	288	0.30 (0.04-2.60)
	No hospital discharge planning	4	661	1.00 (0.86-1.17)
Whellan et al. 2005 [35] (3)	Discharge planning and disease management with			
	Follow up with cardiologist supervision	4	825	0.6 (0.3-0.9) +++
	Follow up with primary care physician supervision	2	662	1.2 (0.9-1.5) +++
	Telephone follow up	3	730	0.8 (0.7-0.9) +++
Holland et al. 2005 [37] (8)	Multidisciplinary interventions			
	Phone/mailing	9	3,349	0.86 (0.73-1.02)
		2	1,701	
	Hospital/clinic/general practice			0.99 (0.90-1.10)
Phillips et al. 2004 [40] (9)	Comprehensive discharge planning with			
	Clinic follow up / frequent telephone contact	4	765	0.64 (0.32-1.28)
McAlister et al. 2004 [41] (6)	Multidisciplinary team providing care			
	In clinic	7	1,183	0.76 (0.58-1.01)
	By telephone follow-up	10	2,923	0.98 (0.80-1.20)
**Coronary heart disease**				
Heran et al. 2011 [51] (10)	Exercise-based cardiac rehabilitation			
	Follow-up of 6 to 12 months	4	463	0.69 (0.51-0.93)
	Follow-up of 12 months or more	7	2,009	0.98 (0.87-1.11)
McAlister et al. 2001 [52] (6)	Secondary prevention programs	6	4,186	0.84 (0.76-0.94)
**Bronchial asthma**				
McLean et al. 2011 [53] (9)	Telehealthcare (personalized care at a distance)			
	Readmissions within 3 months	2	138	0.91 (0.07-12.7)
	Readmissions within 12 months	4	499	0.25 (0.09-0.66)
Tapp et al. 2007 [54] (10)	Education interventions for adults who attend the emergency room for acute asthma	5	566	0.50 (0.27-0.91)
Tsai et al. 2005 [34] (6)	All types of interventions to improve care for asthma	8	1,876	0.76 (0.60-0.97)+
Gibson et al. 2002 [55] (10)	Self-management education of adults with asthma	12	2,418	0.64 (0.50-0.82)
**Prevention of falls in older people in the community**				
Gillespie et al. 2012 [56] (11)	Outcome: number of people sustaining fractures:			
	Exercise	5	570	0.72 (0.47-1.11)
	Vitamin D (with or without calcium)	10	27,070	0.94 (0.82-1.09)
	Multifactorial intervention after assessment	11	3,808	0.84 (0.67-1.05)
Beswick et al. 2008 [57] (7)	Falls prevention interventions and community based care after hospital discharge	41	20,047	0·94 (0·91–0·97)
**Critically ill patients**				
Kim & Soeken 2005 [38] (7)	Inhospital assessment and education with phone follow-up	1	220	0.34 (0.12-0.94)
**Stroke**				
Fearon et al. 2012 [58] (11)	Early supported discharge services	7	918	1.26 (0.94-1.67)
Shepperd et al. 2009 [59] (10)	Hospital at home early discharge:			
	Patients after a stroke at 3 months	3	179	1.06 (0.47-2.38)
**Orthopedic surgery**				
Handoll et al. 2011 [60] (11)	Improving mobility after surgery for hip fractures:			
	Resistance training – at 12 weeks	1	51	0.78 (0.19-3.14)
	Resistance training – at 12 months	1	51	1.39 (0.59-3.43)
	High dose weight bearing (HRR at 16 weeks)	1	150	0.79 (0.35-1.77)
Khan et al. 2008 [61] (9)	Home-based multidisciplinary rehabilitation programs after joint replacement in chronic arthropathy	1	172	0.84 (0.33-2.14)
**Cancer**				
Smeenk et al. 1998 [62] (6)	Home care for patients with incurable cancer	4	923	0.79 (0.55-1.15)+
**Epilepsy**				
Kim & Soeken 2005 [38] (7)	Inhospital assessment and education with phone follow-up	1	42	0.29 (0.07-1.19)
**Chronic obstructive pulmonary disease**				
Jeppesen et al. 2012 [63] (11)	Hospital at home for acute exacerbations	8	870	0.76 (0.59-0.99)
Puhan et al. 2011 [64] (9)	Respiratory rehabilitation after acute exacerbations	5	250	0.22 (0.08–0.58)
Wong et al. 2011 [10] (11)	Nurses visited patients' homes, provided support, education, and monitoring of health.	5	684	1.01 (0.71-1.44)
Lemmens et al. 2009 [65] (7)	Disease-management interventions.	4	602	0.64 (0.51-0.81) +
Shepperd et al. 2009b [59] (10)	Hospital at home after early discharge	4	357	0.83 (0.61-1.13)
Effing et al. 2007 [66] (11)	Self-management education	8	966	0.64 (0.47-0.89)++++
Adams et al. 2007 [67] (7)	Chronic Care Model Multicomponent intervention	4	716	0.79 (0.66-0.94)
	Self-management	3	325	1.02 (0.66-1.57)
Kim & Soeken 2005 [38] (7)	Inhospital assessment and education with phone follow-up	1	66	1.00 (0.02-51.9)
**Patients with chronic disease or geriatric patients**				
Conroy et al. 2011 [68] (9)	Comprehensive geriatric assessment at hospital aimed at rapid discharge with varying degrees of community support	5	2,287	0.95 (0.83–1.08)
Vázquez & Martines 2011 [69](6)	Inhospital and at home medication reconciliation to prevent adverse events	2	1,259	0.87 (0.63-1.19)
Elkan et al. 2001 [70] (10)	Home visiting programs that offer health promotion and preventive care to older people.	6	2,743	0.95 (0.80-1.09)
Shepperd et al. 2009 [59] (10)	Hospital at home early discharge: Older patients with a mix of conditions	5	969	1.35 (1.03-1.76)
Shepperd et al. 2009 [71] (8)	Avoiding hospital admission through provision of hospital care at home	3	416	1.49 (0.96–2.33)
Latour et al. 2007 [72] (7)	Nurse-led case management for ambulatory complex patients in general health care	5	2,395	0.80 (0.60-1.09)+
Kripalani et al. 2007 [73] (5)	Interventions to enhance medication adherence	4	670	0.76 (0.38-1.49)+
Royal et al. 2006 [74] (10)	Pharmacists-led interventions in primary care to reduce medication related adverse events	9	13,132	0.92 (0.80-1.04)
Kim & Soeken 2005 [38] (7)	Inhospital assessment of frail patients with follow up by phone and home visits	3	1,458	0.97 (0.75-1.19)
Parker et al. 2002 [18] (9)	Discharge arrangements in hospital and/or in the community after discharge from hospital care.			
	Both in hospital and in the patient’s home	15	*	0.83 (0.69-1.00)
	Patient’s home only	10	*	0.80 (0.61-1.03)
	Patient education and home follow up.	5	*	0.67 (0.57-0.78)
Mitchell et al. 2002 [75] (4)	Primary medical practitioner involvement with a specialist team	1	364	1.20 (0.86-1.69)+
Hyde et al. 2000 [76] (6)	Supported discharge after acute admission of older patients. Home visits, with or without rehabilitation, commencing 1 week after discharge.	6	916	0.90 (0.77-1.04)+
Stuck et al. 1993 [30] (6)	Comprehensive geriatric assessment.			
	Home assessment service	7	5,240	0.84 (0.73-0.96)
	Hospital and home assessment service	3	847	1.03 (0.56-1.90)
	Outpatient assessment service	4	999	1.24 (0.89-1.73)

HRR – Hospital readmission rates. RCT- Randomized controlled trial.

RR – risk ratio. OR – odds ratio. CI – confidence intervals. ER - Referrals to emergency department.

* not given. + Datum not given; recalculated by the authors of the present meta-review.

++ Risk of sustaining a fracture after falling. +++ Derived from reported figure.

++++ Readmissions for discharge diagnosis only.

### Interventions before hospital discharge

The effect of inhospital interventions on subsequent HRR has been the subject of 11 meta-analyses [14,18,23-31] (Table 1). A single meta-analysis [25] indicated that the implementation of *care pathways for stroke* (1 RCT) reduced HRR by 85%. On the other hand, most other meta-analyses have indicated that *multidisciplinary care* plans in patients with specific disorders (2 meta-analyses, 6 and 3 RCTs) [23,24], *inhospital geriatric case management units* (5 meta-analyses, 1–8 RCTs) [26-30], and *inhospital pharmacological consultations* (1 meta-analysis, 8 RCTs) [31] had no significant effect on HRR. *Geriatric consultation and assessment programs* (3 meta-analyses, 1,3 and 10 RCTs) [18,26,30] produced inconsistent results.

Two meta-analyses of the effect of *discharge planning* similarly produced inconsistent results. The first one included 12 RCTs reported in 1987–2009 that examined the effect of discharge planning prior to leaving hospital, and excluded studies that did not separate the effects of discharge planning from provision of care after discharge from hospital [14]. This meta-analysis revealed a significant 15% average reduction in HRR. The second one [18] reviewed a total of 35 RCTs reported in 1972–1995. Of these, 6 tested discharge planning interventions before hospital discharge only, and they did not detect a significant effect on HRR.

The findings of 11 systematic reviews that synthesized their data using methods other than meta-analyses are presented in Additional file 7: Appendix 7.

### Interventions in the community, with or without hospital discharge planning

Meta-analyses of the effect of interventions in the community [32-76] (Table 2) have indicated that *disease management* programs significantly reduced HRR in patients with heart failure, coronary heart disease and bronchial asthma. In patients with *heart failure*, interventions that included *home care* were almost consistently associated with a reduction in HRR by 12-75%[32-42]. Specifically, *pharmacist care* (1 meta-analysis, 11 RCTs) [45] and *telemonitoring or structured telephone support* (1 meta-analysis, 19 RCTs) [46] reduced HRR by 9-29%. On the other hand, 6 meta-analyses (2–11 RCTs) [32,33,37,40,49,50] found no significant reduction in HRR after *non-structured telephone contact* or *clinic follow-up* of patients with heart failure.

In patients with *coronary heart disease*, secondary prevention programs reduced hospital admissions by 16% (1 meta-analysis, 6 RCTs) [52]. Community care of patients with *bronchial asthma* (3 meta-analyses, 2 – 12 RCTs) [34,53,55] reduced HRR by 9-75%. Other meta-analyses indicated that *exercise training* prevented falls in older people (1 meta-analysis, 5 RCTs) [56], and *inhospital assessment, education and phone follow-up* reduced HRR (1 RCT) [38]. None of the community interventions in patients with *stroke* (2 meta-analyses, 3 and 5 RCTs) [58,59], *hip fractures* (1 meta-analysis, 2 RCTs) [60], *cancer* (1 meta-analysis, 4 RCTs) [62] and epilepsy (1 RCT) [38] had any significant effect on HRR.

Meta-analyses of community interventions in patients with *chronic obstructive pulmonary disease* yielded inconsistent results. Specifically, 4 meta-analyses found a significant reduction in HRR after disease-management interventions [65] (4 RCTs), self-management education [66] (8 RCTs), a multicomponent intervention [67] (4 RCTs), respiratory rehabilitation after acute exacerbations [64] (5 RCTs) and hospital at home for patients with acute exacerbations [63] (8 RCTs). On the other hand, no significant effect on HRR has been found after nursing home care [10] (5 RCTs), hospital at home after early discharge [59] (4 RCTs), self-management [67] (3 RCTs) and inhospital assessment and education with phone follow-up [38] (1 RCT).

Community interventions in *unselected chronic and elderly patients* similarly yielded inconsistent results. Specifically, meta-analyses have indicated a significant reduction in HRR after comprehensive geriatric assessment with a home assessment service (1 meta-analysis, 7 RCTs) [30], discharge arrangements, both inhospital and in the patient’s home (1 meta-analysis, 15 RCTs) [18] and patient education and home follow up (1 meta-analysis, 5 RCTs) [18]. On the other hand, no significant effect on HRR has been found after pharmacists-led interventions for medication reconciliation or for enhancing medication adherence (3 meta-analyses, 2,4 and 9 RCTs) [69,73,74]; hospital-at–home interventions (2 meta-analyses, 3 and 5 RCTs) [59,71]; and in single meta-analyses of inhospital management of frail patients with home follow up (3 RCTs) [38], nurse-led case management for ambulatory complex patients (5 RCTs) [72], and supported discharge after acute admissions of unselected older patients, with home visits after discharge (6 RCTs) [76].

The findings of 36 systematic reviews that synthesized their data using methods other than meta-analyses are presented in Additional file 8: Appendix 8.

## Discussion

### Summary of main findings and conclusions

Almost all meta-analyses that scored 7 or more on the AMSTAR scale indicated that home or community care of patients with heart failure, coronary heart disease and bronchial asthma led to a 12-75% reduction of HRR. This finding suggests that, if future research confirms that efforts to reduce HRR do not adversely affect other patients’ outcomes, all-cause HRR of patients with these disorders may be considered as valid indicators of the quality of medical care in the community.

On the other hand, systematic reviews of the effect of *inhospital* interventions on HRR produced inconsistent findings. One meta-analysis [14] indicated that discharge planning reduced all-cause HRR by an average of 15%. This finding is consistent with the conclusion of Phillips et al. [50] that programs with hospital discharge planning had better patient outcomes than those without. However, it is at odds with the findings of an earlier meta-analysis [18], and of a 2007 meta-review by Mistiaen et al. [13], which found “only limited evidence that hospital discharge interventions have an impact on health care use after discharge”. Therefore, pending the results of future studies, the validity of all-cause HRR as a quality indicator of hospital care is limited. While HRR may be useful for *internal* monitoring of hospital care, their use as *publicly reported* quality indicators may penalize hospitals without reason, thereby violating the requirement that quality indicators should have minimal or no unintended adverse consequences [77].

Additional findings pertain to the optimal management of patients with chronic disease in the community. In patients with heart failure, combinations among discharge planning, patient education, home visits, self-management, telemonitoring, structured telephone support, exercise training and pharmacist care reduced HRR, while telephone or outpatient clinic follow-up did not. Similarly, in patients with coronary heart disease, secondary prevention and rehabilitation programs reduced HRR, while nurse-led coronary heart disease clinics did not. The different effects of telephone / clinic follow-up and other forms of care may be fortuitous; however, as already suggested by Sochalski et al. [78], it may be due to the difference between the individualized care provided at the patient's home, and the relatively impersonal care provided in clinics or by telephone; alternatively, it stands to reason that patients with heart failure, who are randomized between usual care and home care, are more severely sick, and therefore, benefit from any type of care more than those, who are randomized between usual care and telephone / clinic follow-up.

Systematic reviews of the effect of interventions in patients with other chronic diseases either failed to detect significant effect on HRR or produced inconsistent findings. For example, *inhospital* pharmacological consultations, and *community* interventions in patients with stroke, hip fractures, and unselected chronic diseases had no effect on HRR, while hospital discharge planning and community interventions in patients with chronic obstructive pulmonary disease had an inconsistent effect on HRR.

### Study limitations

Our study has four major limitations. First, as we already noted earlier, HRR are not the most important patient-related outcome of health care. For example, *discharge planning* makes sense and, therefore, should be adopted as a standard for hospital care, despite its uncertain effect on HRR. Similarly, *hospital at home for patients with acute exacerbations of chronic pulmonary disorders* has been shown to reduce costs, despite its lack of effect on HRR. Therefore, even if a given intervention fails to reduce HRR, its continuing implementation may still improve other patient outcomes, such as mortality, satisfaction with care and cost-effectiveness. On the other hand, the assumption that HRR reflect patient wellbeing may be erroneous. Interventions aimed at reducing HRR, such as education for self-management, may compromise patients' health by reducing also justified readmissions, and hence the need for further studies of the effect of interventions on patient outcomes. Indeed, a recent RCT comparing HRR of patients with chronic obstructive pulmonary disease, who had usual care and a comprehensive management program combining education, an action plan for identification and treatment of exacerbations and scheduled telephone calls, found that the intervention was associated with an unanticipated excess of HRR and mortality [79].

Second, the search methods that we used did not fully adhere to state of the art recommendations, and we may have failed to identify all published systematic reviews of the effect of clinical interventions on HRR. However, while we agree that, in systematic reviews of *primary* studies, the search should be as wide as possible, we concur with the view by Smith et al. [15] that "in systematic reviews of *reviews*, the searches may be limited to databases specific to systematic reviews, such as the Cochrane Database of Systematic Reviews and the Database of Abstracts of Reviews of Effects, and that … limiting the search to period from the early 1990s onwards is likely to identify all but the very small minority of systematic reviews conducted before then” [15]. We believe that our possible failure to identify all published systematic reviews of the effect of clinical interventions on HRR does not invalidate our two main conclusions, namely, the consistency in the observed reduction in HRR of patients with heart failure and bronchial asthma by community care, and the inconsistent effect of inhospital and community interventions in other patients.

Furthermore, the limited sensitivity of search strategies is shared by many, if not most, reviews: it has been pointed out that the systematic reviews of the same topic by the Cochrane Collaboration and the Task Force on Community Preventive Services differ not only in methods of synthesis of the results, but also in the number of identified studies, with an only limited overlap between sets of included studies [80]. Indeed, our meta-review identified systematic reviews with similar objectives, covering similar periods of time that had retrieved different numbers of primary RCTs. For example, three systematic reviews of the effect of community care of patients with heart failure, covering the years 1966–2003, by Gonseth et al. 2004 [39], Phillips et al. 2004 [40], and McAlister et al. 2004 [41] identified 27, 18, and 29 meta-analyses, respectively (Table 1). This variability was probably due to different search strategies and criteria for exclusion / inclusion of primary studies. In the particular case of these three reviews, the authors reached similar conclusions, despite the different numbers of included meta-analyses. Yet, in other cases, this variability may partly explain different conclusions of apparently similar systematic reviews.

The third limitation of our overview was the use of the same primary RCTs in more than one systematic review. This may have overestimated the consistency of the conclusions of the individual systematic reviews, thereby introducing a bias in the opposite direction. Here again, the studies by Gonseth et al. 2004 [39], Phillips et al. 2004 [40], and McAlister et al. 2004 [41] provide an example. These meta-analyses included 4400, 3304 and 5132 patients respectively; of these, as many as 2037 were shared by all three meta-analyses.

Fourth, even within the defined types of interventions listed in the methods section, there was a wide heterogeneity in methods of implementation. Furthermore, many primary RCTs that were included in individual systematic reviews tested the effect of *combinations* of interventions (e.g., discharge planning *and* phone follow-up), thereby precluding conclusions regarding the efficacy of *single* interventions. This variability detracted from the homogeneity of the RCTs included in the various reviews and from the validity of the various approaches to averaging the outcomes, and may have contributed to the inconsistency among the conclusions of individual reviews.

Here again, the studies by Gonseth et al. 2004 [39], Phillips et al. 2004 [40], and McAlister et al. 2004 [41] provide an example. The meta-analysis by Gonseth et al. 2004 [39] explored the effect of patient education, self-management and support; however the interventions varied with regard to place of initiation of the intervention (hospital or after discharge), type of community care (home or clinic) and duration of the intervention (single visit to 12 months). In the study by Phillips et al. 2004 [40] , 4 of the 18 RCTs addressed the effect of clinic / telephone follow-up, and 14 – the effect of home services that varied from single to multiple visits. In the study by McAlister et al. 2004 [41], 17 of the 29 RCTs assessed the effect of care in outpatient clinics or by telephone follow-up, 8 in a "non-clinic setting" and 4 explored the effect of educational programs.

### Implications for future research

Despite these limitations, the findings of our meta-review suggest two directions for research. First, future study designs should be restricted to RCTs that test the effect of *single* interventions or of the same *combinations* of interventions on patient outcomes. Alternatively, future studies may compare the efficacy of an intervention of undisputed efficacy (e.g., home care of patients with heart failure) with the efficacy of the same intervention combined with a second one (e.g., education for self-management). Hopefully, such studies will clarify the effect of discharge planning without subsequent community care, and of inhospital units for chronically ill / geriatric patients, acute geriatric care and geriatric evaluation and management units, thereby resolving the question whether HRR are a valid quality indicator of hospital care.

Second, the consistencies among the conclusions of systematic reviews with similar objectives are probably partly due to overlapping primary RCTs. However, inconsistent conclusions of systematic reviews of RCTs that test apparently similar interventions (e.g., education for self-management) in apparently similar study populations (e.g., patients with chronic obstructive pulmonary disease) remain unexplained. Therefore, future research should focus on the inconsistencies, rather than consistencies, of the conclusions of individual systematic reviews. These inconsistencies may generate testable hypotheses, such as those that we suggested earlier in order to explain the differences in the efficacy of home care and clinic care for patients with heart failure.

## Competing interests

We hereby declare that we have no potential or actual conflict of interests.

## Authors' contributions

JB conceived the study and performed a preliminary review of the literature. MIT and JB completed the review of the literature and organized the paper as a scientific essay. Both authors read and approved the final manuscript.

## Authors' information

Jochanan Benbassat is a retired physician and, since 1997, a research associate at the Health Policy Research Program of the JDC Meyers-Brookdale Institute. Until 1992, he was a staff physician at the department of Medicine at the Hadassah University Hospital. In 1983, he was appointed Professor of Medicine and chair of Medical Education at the Hebrew University, and in 1992–1997, he was the head of the department of Sociology of Health and chair of Behavioral Sciences in Medicine at the Faculty of Health Sciences in Beer-Sheva.

Mark I Taragin is employed by the National Insurance Institute, Division of Medical Affairs, where he is responsible for Quality Assurance and ongoing computerization of claimant's evaluations. Previously, he worked in the field of computerized medical records, predictive modeling, and pharmaceutical drug development. He has also been a researcher at the JDC-Brookdale Institute and full time faculty at UMDNJ-Rutgers, where he helped start the Division of General Internal Medicine. Throughout his career he has continued to care for patients.

## Supplementary Material

Additional file 1 **Appendix 1.** Search strategies employed in our metareview of systematic reviews of the effect of clinical interventions on hospital readmission rates [81]. Click here for file

Additional file 2 **Appendix 2.** List of excluded full-text papers and of the reasons for their exclusion. Click here for file

Additional file 3 **Appendix 3.** Meta-analyses of controlled trials of the effect of interventions on hospital readmission rates [10,14,18,23-76]. Click here for file

Additional file 4 **Appendix 4.** Other systematic reviews of controlled trials of the effect of interventions on hospital readmission rates [78,82-122]. Click here for file

Additional file 5 **Appendix 5.** Quality assessment of the included meta-analyses of randomized controlled trials of the effect of interventions on hospital readmission rates [10,14,18,23-76]. Click here for file

Additional file 6 **Appendix 6.** Quality assessment of the included other systematic reviews of randomized controlled trials of the effect of interventions on hospital readmission rates [78,82-122]. Click here for file

Additional file 7 **Appendix 7.** Other systematic reviews of randomized controlled trials of the effect of interventions *before* hospital discharge on subsequent hospital readmission rates [82-92]. Click here for file

Additional file 8 **Appendix 8.** Other systematic reviews of randomized controlled trials of the effect of multi-component interventions before and/or after hospital discharge on hospital readmission rates [78,93-122]. Click here for file
