# Supplementary material for: The effect of clinical interventions on hospital readmissions: a meta-review of published meta-analyses
Source: Isr J Health Policy Res. 2013 Jan 23;2:1. doi: 10.1186/2045-4015-2-1 (PMC3557155; doi:10.1186/2045-4015-2-1)
Supplement: Additional file 1 — Appendix 1. Search strategies employed in our metareview of systematic reviews of the effect of clinical interventions on hospital readmission rates [81]. [file 2045-4015-2-1-S1.doc]

Appendix 1. Search strategies employed in our metareview of systematic reviews of the effect of clinical interventions on hospital readmission rates

| Search engine | Key terms | Number of hits |
| --- | --- | --- |
| PaperChase[21] | (a) (readmissions OR patient readmissions) AND (randomized |  |
| (Oldmedline, | controlled trials OR random allocation) AND (review – |  |
| 1960-1965, and | publication type) | 187 |
| Medline, since | (b) (review – publication type OR meta-analysis) AND (home- |  |
| 1966. | care services OR discharge planning OR continuity of patient |  |
|  | care OR patient care team OR health care surveys OR health |  |
|  | services research OR case management OR home care agencies) | 444 |
| Google Scholar | (a) readmissions, randomized controlled trials, meta-analysis | First 270 entries |
|  | (b) readmissions, randomized controlled trials , review | First 270 entries |
| Cochrane | readmissions AND randomized controlled trials | 264 |
| PubMed | readmissions hospital AND randomized controlled trials AND (review OR metaanalysis) | 158 |
| CINAHL | readmissions AND randomized controlled trials | 75 |
