# Supplementary material for: The effect of clinical interventions on hospital readmissions: a meta-review of published meta-analyses
Source: Isr J Health Policy Res. 2013 Jan 23;2:1. doi: 10.1186/2045-4015-2-1 (PMC3557155; doi:10.1186/2045-4015-2-1)
Supplement: Additional file 2 — Appendix 2. List of excluded full-text papers and of the reasons for their exclusion. [file 2045-4015-2-1-S2.doc]

Appendix 2 List of excluded full-text papers and of the reasons for their exclusion.

***Duplicate reviews or updates available***

Cameron I, Crotty M, Currie C, Finnegan T, Gillespie L, Gillespie W, Handoll H, Kurrle S, Madhok R, Murray G, Quinn K, Torgerson D. Geriatric rehabilitation following fractures in older people: a systematic review. Health Technol Assess. 2000; 4: 1–111.

Clark RA, Inglis SC, McAlister FA, Cleland JGF, Stewart S. Telemonitoring or structured telephone support programmes for patients with chronic heart failure: systematic review and meta-analysis. BMJ, 2007; 334: 942.

Crotty M, Unroe K, Cameron ID, Miller M, Ramirez G, Couzner L. Rehabilitation interventions for improving physical and psychosocial functioning after hip fracture in older people. Cochrane Database of Systematic Reviews 2010, Issue1. Art.No.: CD007624.

Gates S, Fisher JD, Cooke MW, Carter YH, Lamb SE. Multifactorial assessment and targeted intervention for preventing falls and injuries among older people in community and emergency care settings: systematic review and meta-analysis. BMJ (2008 Jan 19) 336(7636):130–3

Gillespie LD, Robertson MC, Gillespie WJ, Lamb SE, Gates S, Cumming RG, Rowe BH. Interventions for preventing falls in older people living in the community. Cochrane Database of Systematic Reviews 2009; (2): CD007146.

Griffiths P, Edwards M, Forbes A, Harris R. Post-acute intermediate care in nursing-led units: a systematic review of effectiveness. Int J Nurs Stud 2005; 42: 107–16.

Handoll HH, Cameron ID, Mak JC, Finnegan TP. Multidisciplinary rehabilitation for older people with hip fractures. Cochrane Database Syst Rev 2009; (4):CD007125.

Inglis SC, Clark RA, McAlister FA, Ball J, Lewinter C, Cullington D, Stewart S, Cleland JG. Structured telephone support or telemonitoring programmes for patients with chronic heart failure. Cochrane Database Syst Rev 2010 (8): CD007228

Klersy C, De Silvestri A, Gabutti G, Regoli F, Auricchio A. A meta-analysis of remote monitoring of heart failure patients. Journal of the American College of Cardiology. 2009; 54:1683–1694

Langhorne P, Taylor G, Murray G, Dennis M, Anderson C, Bautz-Holter E, Dey P, Indredavik B, Mayo N, Power M, Rodgers H, Ronning OM, Rudd A, Suwanwela N, Widen-Holmqvist L, Wolfe C. Early supported discharge services for stroke patients: a meta-analysis of individual patients' data. Lancet 2005; 365: 501–6.

Luttik ML, Jaarsma T, Moser D, Sanderman R, van Veldhuisen DJ. The importance and impact of social support on outcomes in patients with heart failure: an overview of the literature. J Cardiovasc Nurs (2005 May-Jun) 20(3):162–9

McAlister FA, Lawson FM, Teo KK, Armstrong PW. A systematic review of randomized trials of disease management programs in heart failure. Am J Med 2001; 110: 378–84.

McLean S, Chandler D, Nurmatov U, Liu JLY, Pagliari C, Car J, Sheikh A. Telehealthcarefor asthma. Cochrane Database ofSystematic Reviews2010, Issue 10. Art.No.:CD007717.

Monninkhof E, van der Valk P, van der Palen J, van Herwaarden C, M R Partridge, G Zielhuis. Self-management education for patients with chronic obstructive pulmonary disease: a systematic review.Thorax 2003; 58: 394–398.

Outpatient Service Trialists: Therapy-based rehabilitation services for stroke patients at home. *Cochrane Database Syst* Rev 2003: CD002925.

Puhan MA, Scharplatz M, Trooster T, Steurer J. Respiratory rehabilitation after acute exacerbation of COPD may reduce risk for readmissions and mortality – a systematic review. Resp Res 2005; 6: 54–66.

Ram FSF, Wedzicha JA, Wright J, Greenstone M. Hospital at home for patients with acute exacerbations of chronic obstructive pulmonary disease: systematic review of evidence.BMJ 2004; 329: 315

Rotter T, Kugler J, Koch R, Gothe H, Twork S, van Oostrum JM, Steyerberg EW. A systematic review and meta-analysis of the effects of clinical pathways on length of stay, hospital costs and patient outcomes. BMC Health Services Research 2008, 8:265

Shepperd S, Doll H, Angus RM, Clarke MJ, Iliffe S, Kalra L, Ricauda NA, Wilson AD. Admission avoidance hospital at home. Cochrane Database Syst Rev 2008; (4): CD007491.

Taylor S, Bestall J, Cotter S, Falshaw M, Hood S, Parsons S, Wood L, Underwood M. Clinical service organisation for heart failure. Cochrane Database Syst Rev 2005; (2): CD002752.

***Studies of the effect of disease specific diagnostic or treatment interventions on hospital readmissions***

Choudhry HK, Singh JM, Barolet A, Tomlinson GA, Detsky AS. How should patients with unstable angina and non-ST segment elevation myocardial infarction be managed? A meta-analysis of randomized trials. The American Journal of Medicine (2005) 118, 465–474

Rhew DC, Tu GS, Ofman J, Henning JM, Richards MS, Weingarten SR. Early switch and early discharge strategies in patients with community-acquired pneumonia: a meta-analysis. Arch Intern Med 2001; 161: 722–7.

Wilt TJ, Niewoehner D, MacDonald R, Kane RL. Management of Stable Chronic Obstructive Pulmonary Disease: A Systematic Review for a Clinical Practice Guideline. Ann Intern Med. 2007; 147: 639–653.

***Prediction models of hospital readmissions, determinants of preventable readmissions, commentaries and state of the art papers***

Bahadori B, FitzGerald JM. Risk factors of hospitalization and readmission of patients with COPD exacerbation – systematic review. International Journal of COPD 2007:2(3) 241–251

Church J, Goodall S, Norman R, Haas M. An economic evaluation of community and residential aged care falls prevention strategies in NSW. N S W Public Health Bull 2011;22: 60–8.

Hickey J; Grotta J. What is the Role of Stroke Units in Overall Care? Disease Management & Health Outcomes 1999; 6: 193–202.

Kansagara D, Englander H, Salanitro A, Kagen D, Theobald C, Freeman M, Kripalani S. Risk prediction models for hospital readmission: a systematic review. JAMA 2011;306: 1688–1698.

Konstam MA. Home monitoring should be the central element in an effective program of heart failure disease management. In: Circulation (2012 Feb 14) 125(6):820–7

Konstam MA; Konstam V. Heart failure disease management a sustainable energy source for the health care engine. Journal of the American College of Cardiology 2010; 56: 379–81.

Lichtman JH, Leifheit-Limson EC, Jones SB, Watanabe E, Bernheim SM, Phipps MS, Bhat KR, Savage SV, Goldstein LB.Predictors of hospital readmission after stroke: a systematic review. Stroke 2010; 41: 2525–33.

Rosenberg AL, Watts C. Patients readmitted to ICUs: a systematic review of risk factors and outcomes. Chest 2000; 118: 492–502.

Scott I. Preventing the rebound: improving care transition in hospital discharge processes. Australian Health Review, 2010, 34, 445–451

Thompson PD, Buchner D, Piña IL, Balady GJ; Williams MA, Marcus BH, Berra K, Blair SN, Costa F, Franklin B, Fletcher GF, Gordon NF, Pate RR, Rodriguez BL, Yancey AK, Wenger NK. Exercise and Physical Activity in the Prevention andTreatment of Atherosclerotic Cardiovascular Disease. Arterioscler Thromb Vasc Biol 2003; 23: e42-e49.

Vest JR, Gamm LD, Oxford BA, Gonzalez MI, Slawson KM. Determinants of preventable readmissions in the United States: a systematic review. Implement Sci 2010; 5: 88–92.

Wells JL, Seabrook JA, Stolee P, Borrie MJ, Knoefel F. State of the Art in Geriatric Rehabilitation. Part I: Review of Frailty and Comprehensive Geriatric Assessment. Arch Phys Med Rehabil 2003; 84: 890–97

***Reviews that failed to identify any eligible studies in the literature search***

NgL, Khan F. Multidisciplinary care for adults with amyotrophic lateral sclerosis or motor neuron disease. Cochrane Database of Systematic Reviews 2009, Issue 4. Art. No.: CD007425.

Ward D, Drahota A, Gal D, Severs M, Dean TP.Care home versus hospital and own home environments for rehabilitation of older people. Cochrane Database Syst Rev 2008; (4): CD003164.

***Interventions targeted at care providers rather than at patients***

Butler M, Collins R, Drennan J, Halligan P, O’Mathúna DP, Schultz TJ, Sheridan A, Vilis E. Hospital nurse staffing models and patient and staff-related outcomes. Cochrane Database of Systematic Reviews 2011, Issue 7. Art. No.: CD007019.

Comondore WR, Devereaux PJ, Zhou Q, Stone SB, Busse JW, Ravindran NC, Burns KE, Haines T, Stringer B, Cook DJ, Walter SD, Sullivan T, Berwanger O, Bhandari M, Banglawala S, Lavis JN, Petrisor B, Schu¨nemann H, Walsh K, Bhatnagar N, Guyatt GH. Quality of care in for-profit and not-for-profit nursing homes:systematic review and meta-analysis. BMJ 2009; 339: B2732.

Eastwood AJ, Sheldon TA. Organisation of asthma care: what difference does it make? A systematic review ofthe literature. Quality in Health Care 1996;5:134–143

Esmonde L, McDonnell A, Ball C, Waskett C, Morgan R, Rashidian A, Bray K, Adam S, Harvey S. Investigating the effectiveness of critical care outreach services: a systematic review. Intensive Care Med (2006) 32:1713–1721

Martin JS, Ummenhofer W, Manser T, Spirig R. Interprofessional collaboration among nurses and physicians: making a difference in patient outcome. Swiss Med Wkly 2010; 140: w13062.

Moonesinghe SR, Lowery J, Shahi N, Millen A, Beard JD. Impact of reduction in working hours for doctors in training on postgraduate medical education and patients' outcomes: systematic review BMJ 2011; 342: d1580

Motamedi SM, Posadas-Calleja J, Straus S, Bates DW, Lorenzetti DL, Baylis B, Gilmour J, Kimpton S, Ghali WA. The efficacy of computer-enabled discharge communication interventions: a systematic review. BMJ Quality & Safety 2011; 20: 403–15.

Shaw C, McNamara R, Abrams K, Cannings-John R, Hood K, Longo M, Myles S, O'Mahony S, Roe B, Williams K. Systematic review of respite care in the frail elderly. Health Technology Assessment 2009; 13: 1–246.

Thomas LH, McColl E, Cullum N, Rousseau N, Soutter J, Steen N Effect of clinical guidelines in nursing, midwifery, and the therapies: a systematic review of evaluations. Qual Health Care 1998; 7: 183–91

Urquhart C, Currell R, Grant MJ, Hardiker NR Nursing record systems: effects on nursing practice and healthcare outcomes. Cochrane Database Syst Rev 2009; (1): CD002099

Wensing M, Wollersheim H, Grol R. Organizational interventions to implement improvements in patient care: a structured review of reviews. Implementation Science 2006, 1:2

***Studies that did not include or specify RCTs with readmissions among the outcomes of interest***

Allard P, Maunsell E, Labbé J, Dorval M. Educational interventions to improve cancer pain control: a systematic review. J Palliat Med 2001 Summer; 4: 191–203.

Ashworth NL, Chad KE, Harrison EL, Reeder BA, Marshall SC. Home versus center based physical activity programs in older adults. Cochrane Database Syst Rev (2005)(1):CD004017

Aubin M, Giguère A,Martin M, Verreault R, Fitch MI, Kazanjian A, CarmichaelPH. Interventions toimprove continuity of care in the follow-up of patients with cancer. Cochrane Database of Systematic Reviews 2012, Issue 7. Art. No.: CD007672.

Bettger JAP, Stineman MG. Effectiveness of Multidisciplinary Rehabilitation Services in Postacute Care: State-of-the-Science. A Review. Arch Phys Med Rehabil 2007; 88: 1526–34.

Bodenheimer T, Wagner EH, Grumbach K. Improving Primary Care for Patients With Chronic IllnessThe Chronic Care Model, Part 2. JAMA 2002; 288(15):1909–1914

Brady BK, McGahan L, Skidmore B. Systematic review of economic evidence on stroke rehabilitation services. International Journal of Technology Assessment in Health Care 2005 21: 15–21.

Clark AM, Hartling L, Vandermeer B, McAlister FA. Meta-Analysis: Secondary Prevention Programs for Patients with Coronary Artery Disease. Ann Intern Med. 2005;143:659–672.

Cole MG. The impact of geriatric post-discharge services on mental state. Age & Ageing 2001; 30: 415–8.

Dalal HM, Zawada A, Jolly K, Moxham T, Taylor RS. Home based versus centre based cardiac rehabilitation: Cochrane systematic review and meta-analysis. BMJ 2010;340:b5631

de Belvis AG, Pelone F, Biasco A, Ricciardi W, Volpe M. Can primary care professionals' adherence to Evidence Based Medicine tools improve quality of care in type 2 diabetes mellitus? A systematic review. Diabetes Res Clin Pract 2009; 85: 119–31.

Ellis G, Langhorne P. Comprehensive geriatric assessment for older hospital patients. British Medical Bulletin 2005; 71: 45–59

Esmonde L, McDonnell A, Ball C, Waskett C, Morgan R, Rashidian A, Bray K, Adam S, Harvey S. Investigating the effectiveness of critical care outreach services: a systematic review. Intensive Care Med 2006; 32: 1713–21.

Forster A, Young J, Lambley R, Langhorne P Medical day hospital care for the elderly versus alternative forms of care. Cochrane Database Syst Rev 2008; (4):CD001730.

Frich LM. Nursing interventions for patients with chronic conditions. J Adv Nurs 2003; 44: 137–53

Hall S, Kolliakou A, Petkova H, Froggatt K, Higginson IJ. Interventions for improving palliative care for older people living in nursing care homes. Cochrane Database Syst Rev 2011; (3): CD007132.

Hedrick SC, Koepsell TD, Inui T. Meta-analysis of home-care effects on mortality and nursing-home placement. Med Care 1989;27: 1015–26.

Hillier S, Inglis-Jassiem G. Rehabilitation for community-dwelling people with stroke: home or centre based? A systematic review. Int J Stroke (2010 Jun) 5(3):178–86

Hoffman BM, Papas RK, Chatkoff DK, Kerns RD Meta-analysis of psychological interventions for chronic low back pain. Health Psychol 2007; 26: 1–9.

Huss A, Stuck AE, Rubenstein LZ, Egger M, Clough-Gorr KM Multidimensional preventive home visit programs for community- dwelling older adults: a systematic review and meta-analysis of randomized controlled trials. J Gerontol A Biol Sci Med Sci 2008; 63: 298–307.

Huss A, Stuck AE, Rubenstein LZ, Egger M, Clough-Gorr KM. Multidimensional preventive home visit programs for community- dwelling older adults: a systematic review and meta-analysis of randomized controlled trials. J Gerontol A Biol Sci Med Sci (2008 Mar) 63(3):298–307

Hysong SJ Meta-analysis: audit and feedback features impact effectiveness on care quality. Med Care 2009; 47: 356–63.

Johnson A, Sandford J, Tyndall J. Written and verbal information versus verbal information only for patients being discharged from acute hospital settings to home. Cochrane Database of Systematic Reviews, 2003 (4)

Jolly K, Taylor RS, Lip GYH, Stevens A. Home-based cardiac rehabilitation compared with centre-based rehabilitation and usual care: A systematic review and meta-analysis International Journal of Cardiology 2006; 111: 343 – 351.

Lacasse Y, Wong E, Guyatt GH, King D, Cook DJ, Goldstein RS. Meta-analysis of respiratory rehabilitation in chronic obstructive pulmonary disease. Lancet 1996;348: 1115–9.

Maric B, Kaan A, Ignaszewski A, Lear SA. A systematic review of telemonitoring technologies in heart failure. European Journal of Heart Failure (2009) 11, 506–517.

Mehta SP, Roy JS. Systematic review of home physiotherapy after hip fracture surgery. J Rehabil Med 2011; 43: 477–480

Meyer TJ, Mark MM Effects of psychosocial interventions with adult cancer patients: a meta-analysis of randomized experiments. Health Psychol 1995; 14: 101–8.

de Morton NA, Keating JL. The effect of exercise on outcomes for older acute medical inpatients compared with control or alternative treatments: a systematic review of randomized controlled trials. Clinical Rehabilitation 2007; 21: 3–16.

Neubeck L, Redfern J, Fernandez R, Briffa T, Bauman A, Freedman SB. Telehealth interventions for the secondary prevention of coronary heart disease: a systematic review. Eur J Cardiovasc Prev Rehabil 2009; 16: 281–9.

Ofman JJ, Badamgarav E, Henning JM, Knight K, Gano AD Jr, Levan RK, Gur-Arie S, Richards MS, Hasselblad V, Weingarten SR. Does disease management improve clinical and economic outcomes in patients with chronic diseases? A systematic review. Am J Med 2004; 117: 182–92.

Oliver D, Connelly JB, Victor CR, Shaw FE, Whitehead A, Genc Y, Vanoli A, Martin FC, Gosney MA. Strategies to prevent falls and fractures in hospitals and care homes and effect of cognitive impairment: systematic review and meta-analyses. BMJ 2007; 334: 82.

Patterson CJ, Mulley GP. The effectiveness of predischarge home assessment visi ts: a systematic review. Clinical Rehabilitation 1999; 13: 101–104

Post PN, Wittenberg J, Burgers JS. Do specialized centers and specialists produce better outcomes for patients with chronic diseases than primary care generalists? A systematic review. International Journal for Quality in Health Care 2009; 21: 387–396.

Richards KC, Enderlin CA, Beck C, McSweeney JC, Jones TC, Roberson PK. Tailored biobehavioral interventions: a literature review and synthesis. Res Theory Nurs Pract 2007; 21: 271–85.

Rousseaux M, Daveluy W, Kozlowski R. Value and efficacy of early supported discharge from stroke units. Ann Phys Rehabil Med (2009 Apr) 52(3):224–33

Ryan R, Santesso N, Hill S, Lowe D, Kaufman C, Grimshaw J. Consumer-oriented interventions for evidence-based prescribing and medicines use: an overview of systematic reviews. Cochrane Database of Systematic Reviews 2011; (5): CD007768.

Seto E. Cost Comparison Between Telemonitoring and Usual Care of Heart Failure: A Systematic Review. Telemed J E Health. 2008 Sep;14(7):679–86

Stroke Unit Trialists’ Collaboration. Organised inpatient (stroke unit) care for stroke. Cochrane Database Syst Rev 2007; (4): CD000197

Taylor RS, Dalal H, Jolly K, Moxham T, Zawada A. Home-based versus centre-based cardiac rehabilitation. Cochrane Database Syst Rev (2010)(1):CD007130

Taylor RS, Brown A, Ebrahim S, Jolliffe J, Noorani H, Rees K, Skidmore B, Stone JA, Thompson DR, Oldridge N. Exercise-Based Rehabilitation for Patients with Coronary Heart Disease: Systematic Review and Meta-analysis of Randomized Controlled Trials. Am J Med 2004; 116: 682– 692.

Teasell RW, Foley NC, Bhogal SK, Speechley MR. Early supported discharge in stroke rehabilitation. Top Stroke Rehabil (2003 Summer) 10(2):19–33

Vaapio SS, Salminen MJ, Ojanlatva A, Kivelä SL. Quality of life as an outcome of fall prevention interventions among the aged: a systematic review. Eur J Public Health 2009; 19: 7–15.

Weingarten SR, Henning JM, Badamgarav E, Knight K, Hasselblad V, Gano A, Ofman J.Interventions used in disease management programmes for patients with chronic illness—which ones work? Meta-analysis of published reports. *BMJ* 2002; 325: 925.

***Primary studies***

Anderson C, Rubenach S, Mhurchu CN, Clark M, Spencer C, Winsor A. Home or Hospital for Stroke Rehabilitation? Results of a Randomized Controlled Trial. Stroke 2000; 31:1024–1031

Bucknall CE, Miller G, Lloyd SM, Cleland J, McCluskey S, Cotton M, Stevenson RD, Cotton P, McConnachie A. Glasgow supported self-managementtrial (GSuST) for patients with moderate to severe COPD: randomized controlled trial. BMJ 2012; 344: e1060

Castro M, Zimmermann NA, Crocker S, Bradley J, Leven C, Schechtman KB. Asthma intervention program prevents readmissions in high healthcare users. Am J Respir Crit Care Med 2003; 168: 1095–9.

Holland R, Brooksby I, Lenaghan E, Ashton K, Hay L, Smith R, Lee Shepstone L, Lipp A, Daly C, Howe A, Hall R, Harvey I. Effectiveness of visits from community pharmacists for patients with heart failure: HeartMed randomized controlled trial. BMJ 2007; 334: 1098–101

Kimmelstiel C, Levine D, Perry K, Patel AR, Sadaniantz A, Gorham N, Cunnie M, Duggan L, Cotter L, Shea-Albright P, Poppas A, LaBresh K, Forman D, Brill D, Rand W, Gregory D, Udelson JE, Lorell B, Konstam V, Furlong K, Konstam MA. Randomized, controlled evaluation of short- and long-term benefits of heart failure disease management within a diverse provider network: the SPAN-CHF trial. Circulation. 2004 Sep 14;110(11):1450–5.

***Studies restricted to avoidable readmissions***

Yam CH, Wong EL, Chan FW, Wong FY, Leung MC, Yeoh EK. Measuring and preventing potentially avoidable hospital readmissions: a review of the literature. Hong Kong Med J (2010 Oct) 16(5):383–9
