# Supplementary material for: The effect of clinical interventions on hospital readmissions: a meta-review of published meta-analyses
Source: Isr J Health Policy Res. 2013 Jan 23;2:1. doi: 10.1186/2045-4015-2-1 (PMC3557155; doi:10.1186/2045-4015-2-1)
Supplement: Additional file 3 — Appendix 3. Meta-analyses of controlled trials of the effect of interventions on hospital readmission rates [10,14,18,23-76]. [file 2045-4015-2-1-S3.doc]

Appendix 3 Meta-analyses of controlled trials of the effect of interventions on hospital readmission rates.

| Reference | Number of primary studies reviewed | | | | Follow-up (months) | Main conclusions | Years covered (Medline) |
| --- | --- | --- | --- | --- | --- | --- | --- |
|  | Total | Studiesincluding | | | HRR |  |  |
|  |  | RCTs | NRCTs | Other |  |  |  |
| Stuck et al. 1993[31] | 28 | 18 | 5 | 0 | 6-36 | Geriatric assessment and management improves survival and function in older persons | 1979-1992 |
| Smeenk et al. 1998[73] | 8 | 4 | 1 | 0 | 6 | Home care programs for cancer patient had no clear effect on patient outcomes | 1974-1997 |
| Hyde et al. 2000[88] | 9 | , | 0 | 0 | 3-12 | Supporting discharge from hospital to home in older people had no clear effect on patients' outcomes | 1972-1995 |
| McAlister et al. 2001[64] | 12 | 6 | 0 | 0 | 1-48 | Disease management programs improve care, reduce hospital admissions and enhance quality of life in patients with coronary heart disease. | 1966-2000 |
| Elkan et al. 2001 [82] | 15 | 6 | 0 | 0 | 12-36 | Home visits to older people reduce mortality and admission to long term institutional care, but not hospital readmissions | 1966-1997 |
| Parker et al. 2002[18] | 54 | 35 | 0 | 0 | 3-6 | Arrangements for discharging older people, with interventions in hospital and in the patient’s home reduce HRR. | 1984-1999 |
| Mitchell et al. 2002[87] | 7 | 1 | 1 | 0 | 6 | Liaison between GPs and specialists leaves most health outcomes unchanged, but improves function in chronically ill patients. | 1966-2001 |
| Lloyd-Williams et al. 2002[60] | 31 | 1 | 0 | 0 | 13 | Physical training in selected patients with heart failure has positive effects on quality of life. | 1966-2000 |
| Gibson et al. 2002[67] | 36 | 12 | 0 | 0 | * | Self-management education and regular practitioner review reduce hospital admissions of adults with asthma | *-* |
| Kwan and Sandercock 2004[26] | 18 | 1 | 1 | 0 | 1 | Insufficient evidence to support implementation of care pathways for hospital management of acute stroke or stroke rehabilitation. | 1975-2003 |
| Gwadry-Sridhar et al. 2004[54] | 8 | 8 | 0 | 0 | 3-12 | Specific heart failure– targeted interventions decrease HRR but not mortality | 1966-2000 |
| Gonseth et al. 2004[51] | 54 | 27 | 27 | 0 | * | Disease management programs reduced HRR for heart failure or cardiovascular disease | 1966-2003 |
| Phillips et al. 2004[52] | 18 | 18 | 0 | 0 | 3-12 | Discharge planning and postdischarge support for older patients with heart failure reduced HRR and may improve survival. | 1966-2003 |
| McAlister et al. 2004[53] | 29 | 29 | 0 | 0 | 0.25-30 | Multidisciplinary care of patients with heart failure reduce heart failure hospitalizations. | 1966-2003 |
| Kim and Soeken 2005[50] | 12 | 10 | 0 | 0 | 3-12 | Hospital based case management decreases HRR by 6% | 1966-2003 |
| Phillips et al. 2005[62] | 6 | 6 | 0 | 0 | 3-12 | Heart failure disease management in specialist nurse-led clinics is a promising strategy | 1966-2004 |
| Tsai et al. 2005[46] | 112 | 24 | 4 | 0 | 1-36 | Interventions that contain one or more elements of the chronic care model improve clinical outcomes and processes of care for patients with chronic illnesses. | 1998-2003 |
| Whellan et al. 2005[47] | 19 | 15 | 0 | 0 | 3-18 | Disease management programs decrease hospitalizations in patients with heart failure. | 1966-2003 |
| Roccaforte et al. 2005[48] | 33 | 25 | 0 | 0 | 3-22 | Disease management programs reduce mortality and hospitalisations in patients with heart failure | 1980-2004 |
| Holland et al. 2005[49] | 30 | 21 | 0 | 0 | * | Multidisciplinary interventions for heart failure, particularly those delivered at home, reduce HRR and mortality. | *-2004 |
| Kaboli et al. 2006[32] | 36 | 7 | 2 | 0 | 1-3 | Clinical pharmacist services improved care without any effect on HRR | 1985-2005 |
| Royal et al. 2006[85] | 38 | 9 | 4 | 0 | * | Weak evidence that pharmacist-led medication reviews reduce HRR | 1966-2005 |
| Jovicic et al. 2006[56] | 6 | 6 | 0 | 0 | 6-12 | Self-management programs for patients with heart failure decrease HRR for heart failure. | 1966-2005 |
| Mistiaen and Poot 2006[61] | 33 | 2 | 1 | 0 | 1.5 | Some studies found that follow-up telephone calls by hospital health professionals in the first month after discharge from hospital had favourable effects for some outcomes | *-2003 |
| Latour et al. 2007[84] | 10 | 5 | 2 | 0 | 3-6 | Nurse-led case management for ambulatory patients improves patient satisfaction | 1966-2005 |
| Kripalani et al. 2007[85] | 37 | 4 | 0 | 0 | * | Interventions aimed to enhance medication adherence in chronic patients improve adherence but only few other clinical outcomes | 1967-2004 |
| Adams et al. 2007[78] | 32 | 7 | 0 | 0 | 2-12 | Two or more chronic care model components reduced HRR in patients with chronic obstructive lung disease | 1966-2005 |
| Kozak et al. 2007[55] | 26 | 21 | 0 | 0 | 1-12 | Non-pharmacologic treatment featuring face-to-face contact reduces heart failure HRR | 1966-2006 |
| Griffiths et al. 2007[30] | 10 | 3 | 2 | 0 | 1 | Nursing-led units for chronically ill or geriatric patients had significant effect on HRR | 1975-2006 |
| Effing et al. 2007[77] | 14 | 8 | 0 | 0 | 2-12 | Self-management education is associated with reduced HRR in patients with chronic obstructive pulmonary disease | 1985-2006 |
| Tapp et al. 2007[66] | 13 | 5 | 0 | 0 | 6-18 | Education interventions for adults who attend the emergency room for acute asthma reduce HRR | *-2009 |
| Auer et al. 2008[25] | 26 | 3 | 2 | 5 | 1-12 | In-hospital multidimensional interventions after acute coronary syndrome had no effect on HRR. | 1980-2007 |
| Koshman et al. 2008[57] | 12 | 11 | 0 | 0 | 6-12 | Pharmacist care in the treatment of patients with heart failure reduces the risk of all-cause HRR. | *-2007 |
| Beswick et al. 2008[83] | 89 | 41 | 0 | 0 | 6-* | Falls prevention interventions and community based care after hospital discharge prevented bone fracures in old patients | *-2005 |
| Khan et al. 2008[72] | 5 | 1 | 0 | 0 | 3 | Home-based multidisciplinary rehabilitation after joint replacement in chronic arthropathy had no significant effect on HRR | *-2006 |
| Shepperd et al. 2009b[70] | 21 | 12 | 0 | 0 | 3 | Early discharge hospital at home services are not associated with economic benefits or improved health outcomes in patients with various conditions | 1950-2008 |
| Shepperd et al. 2009c[84] | 10 | 3 | 0 | 0 | 3 | Hospital care at home yielded similar outcomes to inpatient care, at a similar or lower cost for selected patients | 1966-2008 |
| Lemmens et al. 2009[76] | 36 | 3 | 1 | 0 | 2-12 | Disease management interventions reduced HRR in patients with asthma or chronic obstructive lung disease | 1995-2008 |
| Baztán et al. 2009[29] | 11 | 3 | 2 | 0 | 1-3 | Care in acute geriatric units had no effect on HRR | 1966-2008 |
| Shepperd et al. 2010[14] | 21 | 11 | 0 | 0 | 1-3 | A structured discharge plan brings about small reductions in HRR for older medical inpatients | 1987-2009 |
| Van Craen et al. 2010[28] | 7 | 2 | 0 | 0 | 12 | Geriatric evaluation and management units had no significant effect on HRR | 1966-2007 |
| Rotter et al. 2010[24] | 27 | 6 | 0 | 0 | 1-6 | In-hospital clinical pathways reduced complications and improved documentation without effect on HRR and mortality. | * |
| Puhan et al. 2011[75] | 9 | 5 | 0 | 0 | 3-18 | Respiratory rehabilitation after exacerbation of chronic obstructive lung disease reduce HRR | 1966-2010 |
| Vázquez & Martines 2011[80] | 4 | 2 | 0 | 0 | * | Medication reconciliation prevent adverse events. Authors’ conclusión that medication reconciliation significantly reduces HRR is not borne out by data | * |
| Inglis et al. 2011[58] | 30 | 19 | 0 | 0 | 3-15 | Telemonitoring and structured telephone support improve outcomes in patients with heart failure | 1966-2008 |
| McLean et al. 2011[65] | 21 | 6 | 0 | 0 | 3-12 | Telehealthcare interventions for patients with asthma reduces HRR | * |
| Wong et al. 2011[10] | 9 | 5 | 0 | 0 | 3-12 | Outreach nursing programs for patients with choronic obstructive lung disease had no significant effect on HRR | *-2009 |
| Handoll et al. 2011[71] | 19 | 2 | * | * | 3,4 | Insufficient evidence to establish the best strategies for enhancing mobility after hip fracture surgery | 1966-2010 |
| Heran et al. 2011 [63] | 47 | 11 | 0 | 0 | 6-120 | Exercise-based cardiac rehabilitation reduced HRR | 1950-2009 |
| Klersy et al. 2011[59] | 21 | 18 | 0 | 0 | 3-18 | Remote patient monitoring was associated with a significantly lower number of HRR of heart failure patients | 2000-2009 |
| Conroy et al. 2011[79] | 5 | 5 | 0 | 0 | 1-12 | Comprehensive geriatric assessment at hospital with varying degrees of community support had no effect on HRR | *-2009 |
| Ellis et al. 2011[27] | 22 | 9 | 0 | 0 | 1.5-12 | Comprehensive geriatric assessment after an emergency admission to hospital had no effect on HRR | *-2010 |
| Jeppesen et al. 2012[74] | 8 | 8 | 0 | 0 | 1-12 | Hospital at home for acute exacerbations of chronic obstructive pulmonary disease reduced HRR | *-2010 |
| Takeda et al. 2012[45] | 25 | 13 | 0 | 0 | 6-12 | Case management, outpatient clinic, or multidisciplinary care, led by a heart failure specialist nurse reduces HRR and mortality in patients with heart failure | 1950-2009 |
| Lambrinou et al. 2012[44] | 19 | 19 | 0 | 0 | 3-35 | Heart failure management programs with nurse-driven pre-discharge interventions and home care reduce HRR | *-2009 |
| Fearon et al. 2012[69] | 14 | 7 | 0 | 0 | 3-12 | Supported discharge for stroke patients reduce longterm dependency but not HRR | 1966-2012 |
| Gillespie et al. 2012[68] | 159 | * | 0 | 0 | 1-60 | The evidence relating to the provision of exercise programs, education programs, medication optimisation, environmental modification or multiple intervention for preventing fractures in elderly persons is inconclusive. | 1946-2012 |

*Not reported
