# Supplementary material for: The effect of clinical interventions on hospital readmissions: a meta-review of published meta-analyses
Source: Isr J Health Policy Res. 2013 Jan 23;2:1. doi: 10.1186/2045-4015-2-1 (PMC3557155; doi:10.1186/2045-4015-2-1)
Supplement: Additional file 4 — Appendix 4. Other systematic reviews of controlled trials of the effect of interventions on hospital readmission rates [78,82-122]. [file 2045-4015-2-1-S4.doc]

Appendix 4 Other systematic reviews of controlled trials of the effect of interventions on hospital readmission rates.

| Reference | Number of primary studies reviewed | | | | Follow-up (months) | Main conclusions | Years covered (Medline) |
| --- | --- | --- | --- | --- | --- | --- | --- |
|  | Total | Studies including | | | HRR |  |  |
|  |  | RCTs | NRCTs | Other |  |  |  |
| Hughes et al. 1997[118] | 20 | 11 | 9 | 0 | 1-48 | Home care of geriatric patients led to a small to moderate reduction in hospital days | 1964-1994 |
| Ferguson & Weinberger 1998[101] | 9 | 7 | 0 | 0 | 3-12 | No strong endorsement of case management programs in primary care | 1985-1997 |
| Griffin 1998[107] | 5 | 2 | 0 | 0 | 12-60 | 1 trial favored hospital outpatient care for diabetic patients; 1 trial favored GP care | * |
| Scott 1999[42] | 24 | 12 | 0 | 0 | 2-36 | Discharge plans, psychogeriatric assessment and home care reduces HRR of elderly patients. | 1984-1998 |
| Parker et al. 2000[37] | 45 | 21 | 0 | 0 | 1-12 | Weak evidence about effectiveness and costs of various care modalities for older patients | 1988-1999 |
| Smith et al. 2001[38] | 4 | 1 | 0 | 0 | * | Home care by outreach nursing for chronic obstructive pulmonary disease had no effect on HRR | * |
| Berendsen et al. 2002[117] | 27 | 12 | 1 | 0 | 1-6 | "Disappointing" reduction in costs to the health services of hospital at home | 1990-2001 |
| Richards & Coast 2003[41] | 15 | 11 | 0 | 0 | 2-24 | Interventions intended to improve access to health and social care for older patients after discharge from hospitals had an inconsistent effect on HRR. | 1966-2000 |
| Balinsky and Muennig 2003[36] | 4 | 4 | 0 | 0 | 3-4 | Inpatient-based multifaceted interventions were effective and inexpensive in reducing HRR. | 1988-2000 |
| Louis et al. 2003[100] | 24 | 4 | 11 | 1 | 6-12 | Telmonitorng of patients with heart failure reduced HRR in all 12 NRCTs but only in 2 of 4 RCTs | 1966-2002 |
| Gustafsson and Arnold 2004[99] | 31 | 18 | 13 | 0 | 3-12 | The majority of the RCTs comparing heart failure clinics using nurse intervention with conventional care have shown either a reduction in HRR or shorter hospitalisations in the intervention group. | *-* |
| Page et al. 2005[102] | 6 | 2 | 0 | 0 | 4-12 | Equivocal evidence about the effect of nurse-led clinic for patients with coronary heart disease on HRR | 1966-2002 |
| Taylor et al. 2005[104] | 9 | 5 | 0 | 0 | 9-12 | Equivocal evidence about the effect of nurse management of chronic obstructive pulmonary disease on HRR | 1980-2005 |
| Hastings et al. 2005[116] | 27 | 4 | 2 | * | * | Discharge planning / comprehensive geriatric assessment and home follow up did not affect HRR in 3 of 4 RCTs in elders discharged from emergency hospital departments | *-* |
| Worrall and Knight 2006[115] | 5 | 2 | 2 | 0 | 18,24 | Continuity of care for older patients in family practice reduced emergency room admissions. | 1970-2005 |
| Göhler et al. 2006[97] | 36 | 32 | 0 | 0 | 3-18 | Disease management programs for patients with heart failure significantly reduced all-cause HRR | 1966-2005 |
| Larsen et al. 2006[109] | 7 | * | 0 | 0 | 3-12 | Early home supported discharge of patients with stroke had no effect on HRR | *-2005 |
| Yu et al. 2006[98] | 21 | 21 | 0 | 0 | 3-12 | Cardiac disease management programs significantly reduced HRR in about half of the reviewed RCTs. | 1995-2004 |
| Martínez et al. 2006[96] | 42 | 4 | 2 | 11 | * | Telemonitoring led to an improved patient follow-up, as well as to reduced HRR and emergency visits. |  |
| Chiu and Newcomer 2007[94] | 16 | 16 | 0 | 0 | 3-12 | Home visits, continuous contact with patients and patient education by specialized nurses reduced HRR in 8 of the 15 trials. | 1996-2006 |
| Smith et al. 2007[93] | 20 | 5 | 1 | 0 | 3-24 | Insufficient evidence supporting shared primary and specialty care in chronic disease management | 1966-2006 |
| Halbert et al. 2007[35] | 11 | 5 | 0 | 0 | 3-12 | Multi-disciplinary rehabilitation after hip fracture reduced mortality but not HRR | *-2005 |
| Ponniah et al. 2007[95] | 7 | 4 | 2 | 0 | 6-12 | A medication management service for patients with heart failure reduced unplanned admissions | 1990-2006 |
| Garcıa-Lizana, Sarrıa-Santamera 2007 [92] | 24 | 6 | 0 | 0 | 6-15 | Equivocal evidence that information / communication technologies, such as the internet and telemedicine, reduce HRR | 1995-2005 |
| Winkel et al. 2008[108] | 17 | 6 | 0 | 0 | 3-6 | Early supported discharge from hospital with nursing, physical, occupational and speech therapy in patients’ homes, had no effect on HRR of patients with stroke | 1980-2005 |
| Hsiao, Boult 2008[114] | 14 | 1 | 3 | 2 | * | Continuity of primary care led to fewer emergent hospital addmissions | 1950-2006 |
| Allen et al. 2009[34] | 7 | 5 | 0 | 0 | * | Integrated care pathways improve quality of care, adherence to guidelines and clinical decision-making but do not reduce HRR. | 1980-2008 |
| Lemmens et al. 2009[33] | 13 | 1 | 3 | 9 | * | Clinical pathways for digestive surgery reduced length of stay but not HRR | 2000-2006 |
| Preyde et al. 2009[40] | 25 | 13 | 2 | 0 | 1-18 | Most studies found that discharge planning reduced HRR in the short term | 1995-2005 |
| Sochalski et al. 2009[91] | 10 | 10 | 0 | 0 | 3-12 | Multidisciplinary teams with in-person communication reduced HRR in patients with heart failure | 1990-2004 |
| Oeseburg et al. 2009[113] | 8 | 6 | 0 | 0 | 12-24 | Cordination of care of impaired older or chronic patients in the community by education, self-management, home visits or telephone contact had no effect on HRR | 1995-2007 |
| Batty 2010[112] | 13 | 7 | 2 | 3 | 1-24 | The most effective models in preventing HRR in older people are provided by teams in the patient’s home | 2000-2009 |
| Ditewig et al. 2010[90] | 19 | 8 | 0 | 0 | 6-12 | Shortcomings of published studies do not permit the validation of the effect of self-management interventions on HRR of patients with heart failure | 1996-2009 |
| Chisholm-Burns et al. 2010[111] | 298 | 35 | * | * | * | Pharmacist-provided patient care had favorable effects on HRR in 18 of 35 studies | 1950-2009 |
| Walters et al. 2010[103] | 5 | 3 | 0 | 0 | 6-12 | Guidelines for self care had no effect on HRR of patients with chronic obstructive lung disease | *-2009 |
| Young and Busgeeth 2010[106] | 13 | 3 | 0 | 0 | * | Home-based nursing improved self-reported knowledge of HIV and medications, self-reported adherence, worry and physical functioning, and reduced HRR in one of three trials. | 1980-2008 |
| Bachman et al. 2010[38] | 17 | 6 | 0 | 0 | 3-12 | Inpatient geriatric rehabilitation has the potential to improve outcomes related to function, admission to nursing homes, and mortality. The range of HRR was similar in intervention and control patients. | 1970-2008 |
| Ahmed and Shannon 2010 [39] | 17 | 5 | 1 | 1 | * | Acute care units for elderly inpatient are associated with reduced cost, length of stay, readmission rates, and enhanced rehabilitation, cognition, function and patient/staff satisfaction. | 1990-2008 |
| Hansen et al. 2011[43] | 43 | 16 | 20 | 7 | 1 | No single intervention before or after hospital discharge reduced 30-day HRR | 1975-2011 |
| Schadewaldt & Schultz 2011[89] | 7 | 1 | 0 | 0 | 120 | Care in nurse-led clinics for patients with coronary heart disease was equivalent to that in other clinics and had no effect on HRR. | 2002-2008 |
| Boyde et al. 2011[119] | 19 | 13 | 0 | 0 | 3-12 | Education of patients with heart failure reduced HRR in 4 of 13 trials | 1998-2008 |
| Smith et al. 2012[110] | 10 | 5 | 0 | 0 | 2-24 | Most trials of interventions designed to improve outcomes in patients with multimorbidity in primary care and community settings did not find evidence for reduced HRR | 1990-2011 |

RCT – randomized controlled trial. NRCT – non-randomized controlled trials. HRR – Hospital readmission rates. OPD – outpatient department. GP – general practice

* - not given
