# Supplementary material for: The effect of clinical interventions on hospital readmissions: a meta-review of published meta-analyses
Source: Isr J Health Policy Res. 2013 Jan 23;2:1. doi: 10.1186/2045-4015-2-1 (PMC3557155; doi:10.1186/2045-4015-2-1)
Supplement: Additional file 5 — Appendix 5. Quality assessment of the included meta-analyses of randomized controlled trials of the effect of interventions on hospital readmission rates [10,14,18,23-76]. [file 2045-4015-2-1-S5.doc]

Appendix 5 Quality assessment of the included meta-analyses of randomized controlled trials of the effect of interventions on hospital readmission rates.

| Reference | AMSTAR criteria | | | | | | | | | | |
| --- | --- | --- | --- | --- | --- | --- | --- | --- | --- | --- | --- |
|  | "A priori" design | >1 data extractors / reviewers | >1 e-sources | Statement of inclusion of studies by publication status | List of excluded studies | Data on participants, interventions and outcomes | Quality assessed | Quality considered | Tests of heterogeneity | Publication bias | Conflict of interests |
| Stuck et al. 1993[31] | + | + |  | + |  | + |  |  | + | + |  |
| Smeenk et al. 1998[73] | + | + | + |  |  | + | + |  |  |  | + |
| Hyde et al. 2000[88] | + | + | + |  |  | + | + |  | + |  |  |
| McAlister et al. 2001[64] | + | + | + |  |  | + |  |  | + |  | + |
| Parker et al. 2002[18] | + | + | + | + | + | + | + |  | + | + |  |
| Elkan et al. 2001[82] | + | + | + | + | + | + | + |  | + | + | + |
| Mitchell et al. 2002[87] | + |  | + |  |  | + | + |  |  |  |  |
| Lloyd-Williams et al. 2002[60] | + | + | + |  |  | + | + |  |  |  |  |
| Gibson et al. 2002[67] | + | + | + | + | + | + | + |  | + | + | + |
| Kwan and Sandercock 2004[26] | + | + | + |  | + | + | + | + | + | + | + |
| Gwadry-Sridhar et al. 2004[54] | + | + | + |  |  | + | + |  | + | + |  |
| Gonseth et al. 2004[51] | + | + | + |  | + | + | + |  | + | + | + |
| Phillips et al. 2004[52] | + | + | + |  |  | + | + | + | + | + | + |
| McAlister et al. 2004[53] | + | + | + |  |  | + | + |  | + |  |  |
| Kim and Soeken 2005[50] | + | + | + |  |  | + | + |  | + | + |  |
| Phillips et al. 2005[62] | + | + | + |  |  | + | + | + | + | + |  |
| Tsai et al. 2005[46] | + |  | + |  |  | + | + |  | + | + |  |
| Whellan et al. 2005[47] | + |  |  |  |  | + |  |  | + |  |  |
| Roccaforte et al. 2005[48] | + | + | + | + |  | + | + | + | + | + |  |
| Holland et al. 2005[49] | + | + | + |  |  | + | + | + | + | + |  |
| Kaboli et al. 2006[32] | + | + | + |  |  | + |  |  |  |  |  |
| Royal et al. 2006[86] | + | + | + | + |  | + | + | + | + | + | + |
| Jovicic et al. 2006[56] | + | + | + |  |  | + | + |  | + | + | + |
| Mistiaen and Poot 2006[61] | + | + | + |  | + | + | + |  | + |  | + |
| Latour et al. 2007[84] | + | + | + |  |  | + | + | + | + |  |  |
| Kripalani et al. 2007[85] | + | + | + |  |  | + |  |  |  |  | + |
| Adams et al. 2007[78] | + | + | + |  |  | + | + |  | + | + |  |
| Kozak et al. 2007[55] | + | + | + |  |  | + | + |  | + |  |  |
| Griffiths et al. 2007[30] | + | + | + |  | + | + | + | + | + |  | + |
| Effing et al. 2007[77] | + | + | + | + | + | + | + | + | + | + | + |
| Tapp et al. 2007[66] | + | + | + | + | + | + | + | + | + | + |  |
| Auer et al. 2008[25] | + | + | + |  |  | + | + |  | + | + |  |
| Koshman et al. 2008[57] | + | + | + |  |  | + | + | + | + |  | + |
| Beswick et al. 2008[83] | + | + | + |  |  | + |  |  | + | + | + |
| Khan et al. 2008[72] | + | + | + |  | + | + | + | + | + |  | + |
| Shepperd et al. 2009[70] | + | + | + | + | + | + | + | + | + |  | + |
| Shepperd et al. 2009[84] | + | + | + |  | + | + | + |  | + |  | + |
| Lemmens et al. 2009[76] | + | + | + |  |  | + | + |  | + |  | + |
| Baztán et al. 2009[29] | + | + | + |  |  | + | + |  | + | + | + |
| Shepperd et al. 2010[14] | + | + | + | + | + | + | + | + | + |  |  |
| Van Craen et al. 2010[28] | + | + | + |  |  | + | + |  | + |  | + |
| Rotter et al. 2010[24] | + | + | + |  | + | + | + | + | + | + | + |
| Vázquez & Martines 2011[80] | + | + | + | + |  | + | + |  |  |  |  |
| Puhan et al. 2011[75] | + | + | + | + | + | + | + |  | + |  | + |
| Inglis et al. 2011[58] | + | + | + | + |  | + | + | + | + | + | + |
| McLean et al. 2011[65] | + | + | + | + |  | + | + |  | + | + | + |
| Wong et al. 2011[10] | + | + | + | + | + | + | + | + | + | + | + |
| Handoll et al. 2011[71] | + | + | + |  | + | + | + |  | + |  | + |
| Heran et al. 2011[63] | + | + | + | + | + | + | + |  | + | + | + |
| Klersy et al. 2011[59] | + | + | + |  |  | + | + |  | + |  | + |
| Conroy et al. 2011[79] | + | + | + |  |  | + | + |  | + | + | + |
| Ellis et al. 2011[27] | + | + | + |  |  | + | + | + | + |  |  |
| Jeppesen et al. 2012[74] | + | + | + | + | + | + | + | + | + | + | + |
| Takeda et al. 2012[45] | + | + | + | + | + | + | + | + | + |  | + |
| Lambrinou et al. 2012[44] | + | + | + |  |  | + | + | + | + | + | + |
| Fearon et al. 2012[69] | + | + | + | + | + | + | + | + | + | + | + |
| Gillespie et al. 2012[68] | + | + | + | + | + | + | + | + | + | + | + |
