# Supplementary material for: The effect of clinical interventions on hospital readmissions: a meta-review of published meta-analyses
Source: Isr J Health Policy Res. 2013 Jan 23;2:1. doi: 10.1186/2045-4015-2-1 (PMC3557155; doi:10.1186/2045-4015-2-1)
Supplement: Additional file 6 — Appendix 6. Quality assessment of the included other systematic reviews of randomized controlled trials of the effect of interventions on hospital readmission rates [78,82-122]. [file 2045-4015-2-1-S6.doc]

Appendix 6 Quality assessment of the included other systematic reviews of randomized controlled trials of the effect of interventions on hospital readmission rates.

| Reference | AMSTAR criteria | | | | | | | | | | |
| --- | --- | --- | --- | --- | --- | --- | --- | --- | --- | --- | --- |
|  | "A priori" design | >1 data extractors / reviewers | >1 e-sources | Statement of inclusion of studies by publication status | List of excluded studies | Data on participants, interventions and outcomes | Quality assessed | Quality considered | Tests of heterogeneity/ appropriate data synthesisor presentation | Publication bias | Conflict of interests |
| Hughes et al. 1997[118] | + | + | + | + |  | + |  |  | + |  |  |
| Ferguson & Weinberger 1998[101] | + |  | + |  |  | + |  |  | + |  |  |
| Griffin 1998[107] | + |  | + |  |  | + |  |  | + |  | + |
| Scott 1999[42] | + |  | + |  |  | + |  |  | + |  |  |
| Parker et al. 2000[37] | + | + | + |  |  | + | + |  | + |  |  |
| Smith et al. 2001[38] | + | + | + | + | + | + | + | + | + |  | + |
| Berendsen et al. 2002[117] | + |  | + |  |  | + |  |  | + |  | + |
| Richards & Coast 2003[41] | + |  | + |  |  | + | + | + |  |  |  |
| Balinsky and Muennig 2003[36] | + |  |  |  |  | + | + | + | + |  |  |
| Louis et al. 2003[100] | + | + | + | + |  | + |  |  | + |  |  |
| Gustafsson and Arnold 2004[99] | + |  |  |  |  | + |  |  | + |  |  |
| Page et al. 2005[102] | + | + | + |  |  | + | + |  | + |  |  |
| Taylor et al. 2005[104] | + | + | + | + |  | + | + | + | + |  | + |
| Hastings et al. 2005[116] | + | + | + |  |  | + | + |  | + |  |  |
| Worrall and Knight 2006[115] | + | + | + |  |  | + | + | + | + |  | + |
| Göhler et al. 2006[97] | + | + |  |  |  | + |  |  | + | + |  |
| Larsen et al. 2006[109] | + |  |  |  |  | + |  |  |  |  |  |
| Martínez et al. 2006[96] | + | + | + |  |  | + | + |  |  |  |  |
| Yu et al. 2006[98] | + |  | + |  |  | + |  |  | + |  | + |
| Chiu and Newcomer 2007[94] | + |  |  |  |  | + |  |  | + |  | + |
| Smith et al. 2007[93] | + | + | + | + | + | + | + | + | + |  | + |
| Halbert et al. 2007[35] | + | + | + |  |  | + | + |  | + |  | + |
| Ponniah et al. 2007[95] | + |  | + |  |  | + | + | + |  |  |  |
| Garcıa-Lizana, Sarrıa-Santamera 2007[92] | + | + | + |  |  | + | + |  | + |  |  |
| Winkel et al. 2008[108] | + |  | + |  |  | + | + |  | + |  |  |
| Hsiao and Boult 2008[114] | + |  |  |  |  | + |  |  |  |  |  |
| Allen et al. 2009[34] | + |  | + | + | + | + | + |  |  |  |  |
| Lemmens et al. 2009[33] | + | + | + |  |  | + |  |  |  |  | + |
| Preyde et al. 2009[40] | + | + | + |  |  | + | + |  | + |  |  |
| Sochalski et al. 2009[91] | + |  |  |  |  | + |  |  | + |  |  |
| Oeseburg et al. 2009 [113] | + | + | + |  |  | + | + |  | + |  |  |
| Batty 2010[112] | + |  | + |  |  | + | + |  | + |  | + |
| Ditewig et al. 2010[90] | + | + | + |  |  | + | + | + | + |  | + |
| Chisholm-Burns et al. 2010[111] | + | + | + |  |  | + | + |  | + | + |  |
| Walters et al. 2010[103] | + | + | + | + | + | + | + | + | + | + | + |
| Young and Busgeeth 2010[106] | + | + | + | + | + | + | + |  | + |  | + |
| Bachman et al. 2010[38] | + | + | + |  | + | + | + | + | + | + | + |
| Ahmed and Shannon 2010[39] | + |  | + |  |  | + |  |  | + |  | + |
| Hansen et al. 2011[43] | + | + | + |  |  | + | + |  | + | + | + |
| Schadewaldt & Schultz 2011[89] | + | + | + | + |  | + | + |  | + |  | + |
| Boyde et al. 2011[119]. | + | + | + |  |  | + |  |  | + |  |  |
| Smith et al. 2012[110] | + | + | + | + | + | + | + |  | + |  | + |
