# Supplementary material for: The effect of clinical interventions on hospital readmissions: a meta-review of published meta-analyses
Source: Isr J Health Policy Res. 2013 Jan 23;2:1. doi: 10.1186/2045-4015-2-1 (PMC3557155; doi:10.1186/2045-4015-2-1)
Supplement: Additional file 7 — Appendix 7. Other systematic reviews of randomized controlled trials of the effect of interventions before hospital discharge on subsequent hospital readmission rates [82-92]. [file 2045-4015-2-1-S7.doc]

Appendix 7 Other systematic reviews of randomized controlled trials of the effect of interventions *before* hospital discharge on subsequent hospital readmission rates.

| Reference  (AMSTAR score) | Intervention | RCTs that reported HRR (n) | Total number of patients | Reported effect of intervention on HRR |
| --- | --- | --- | --- | --- |
| **Disease management programs** | | | | |
| Lemmens et al. 2009[33](6) | Clinical pathways for digestive surgery | 1 | 64 | No significant differences in HRR. |
| Allen et al. 2009[34](6) | Clinical pathways (Multidisciplinary care, monitoring, and evaluation of outcomes) for patients with |  |  | No significant differences in HRR |
|  | Orthopedic surgery | 2 | 111,* |  |
|  | Bronchial asthma | 1 | 251 |  |
|  | Intestinal resection | 1 | 64 |  |
|  | Heart failure | 1 | 132 |  |
| Halbert et al. 2007[35] (7) | Multi-disciplinary rehabilitation after hip fracture | 4 | 1,115 | No differences in HRR. |
|  |  | 1 | 135 | Significantly reduced HRR |
| Balinsky and Muennig 2003[36] (5) | Interventions designed to improve the care of heart failure | 4 | 576 | Significant reduction in HRR or in hospital days by 43 -57% |
| Parker et al. 2000[37](6) | Hip replacement units | 1 | 371 | Significantly reduced HRR |
| Geriatric case management units/ rehabilitation | | | | |
| Bachman et al. 2010[38] (10) | Inpatient rehabilitation specifically designed for geriatric patients | 6 | * | Lower or equal rate of HRR in intervention patients. Similar range of HRR in intervention and control patients. |
| Ahmed and Shannon 2010[39](5) | Acute care units for hospitalized adults older than 65 years. | 3 | * | Reduced HRR or total costs |
|  |  | 2 | * | “Neutral impact on admissions" |
| Preyde et al. 2009[40](6) | Geriatric evaluation and acute care | 1 | * | No differences in HRR. |
| Parker et al. 2000[37](6) | Nurse led geriatric unit | 2 | * | No differences in HRR/days of readmision |
|  | Geriatric evaluation/care | 1 | * | Significantly reduced HRR |
|  |  | 3 | * |  |
|  |  |  |  | No differences in HRR |
| **Geriatric consultations and assessment programs** | | | | |
| Richards & Coast 2003[41](5) | Geriatric consultation | 1 | 132 | Significantly reduced HRR |
|  | Geriatric evaluation and management | 2 | 270 | No difference in HRR |
| Scott 1999[42](4) | Geriatric evaluation and management | 1 | 155 | No differences in use of health facilities |
|  | Geriatric consultations | 1 | 120 | Significantly reduced HRR |
|  |  | 1 | 237 |  |
|  | Psychogeriatric team management |  |  | Significantly reduced HRR |
| **Pharmacological consultations** | | | | |
| Preyde et al. 2009[40](6) | Discharge planning by hospital pharmacist | 1 | * | No significant sifferences in HRR. |
| Discharge planning | | | | |
| Hansen et al. 2011[43] (8) | Discharge planning only | 1 | 835 | Singnificantly reduced HRR by 11% |
|  |  | 1 | 620 | No effect on HRR |
| Preyde et al. 2009[40](6) | Nursing care for prevention of disability and rehabilitation, discharge planning and review of medical care. | 1 | * | No sifferences in HRR. |
| Richards and Coast 2003[41] (5) | Discharge planning aimed at improving access to health and social care. | 2 | 600 | Significantly reduced HRR. |
|  |  | 3 | 828 | No differences in HRR |
| Scott 1999[42] (4) | Discharge planning for heart failure patients | 1 | 282 | Significantly reduced HRR at 3 months |

HRR – Hospital readmission rates. RCT- Randomized controlled trials. *- not given
