# Supplementary material for: The effect of clinical interventions on hospital readmissions: a meta-review of published meta-analyses
Source: Isr J Health Policy Res. 2013 Jan 23;2:1. doi: 10.1186/2045-4015-2-1 (PMC3557155; doi:10.1186/2045-4015-2-1)
Supplement: Additional file 8 — Appendix 8. Other systematic reviews of randomized controlled trials of the effect of multi-component interventions before and/or after hospital discharge on hospital readmission rates [78,93-122]. [file 2045-4015-2-1-S8.doc]

Appendix 8: Other systematic reviews of randomized controlled trials of the effect of multi-component interventions before and/or after hospital discharge on hospital readmission rates

| **Reference**  **(AMSTAR score)** | **Intervention** | **RCTs that reported HRR (n)** | | **Total number of patients** | **Findings** |
| --- | --- | --- | --- | --- | --- |
| **Heart failure** | | | | | |
| Hansen et al. 2011[43](8) | Predischarge patient education and discharge planning with follow-up phone calls and communication with community care providers | 3 | | 283 | No significant differences in HRR |
| Boyde et al. 2011[119](5) | Patient education | 4 | | 503 | Significant reduction in HHR. |
|  |  | 9 | | 5,013 | Non-significant reduction in HHR |
| Schadewaldt & Schultz 2011[89](8) | Nurse-led clinics for cardiac patients | 1 | | 1,073 | No significant differences in HRR |
| Ditewig et al. 2010[90](8) | Self-management interventions | 3 | | 946 | Significant reduction in HHR. |
|  |  | 5 | | 1,203 | Non-significant reduction in HHR. |
| Sochalski et al. 2009[91](3) | Chronic care management | 7 | | 1,429 | Significant reduction of HRR by 27 to 44%. |
|  |  | 3 | | 599 | No effect on HRR. |
| Preyde et al. 2009[40](6) | Transitional care Multidisciplinary nurse discharge planning and home follow-up | 1 | | * | No sifferences in HRR. |
|  |  | 2 | | * | Significantly reduced HRR. |
| Garcıa-Lizana and Sarrıa-Santamera 2007[92] (6) | Use of information / communication technologies (internet, videoconference, interactive media, telemedicine). The patients are receptors of the activity or the source of data. | 2 | | 228 | Significant reduction in HHR. |
|  |  | 2 | | 387 | Non-significant reduction in HHR. |
| Smith et al. 2007[93](10) | Shared care between primary and specialty care in chronic disease management. | 1 | | 127 | Significntly reduced HRR |
| Chiu& Newcomer 2007[94](4) | Nurse-assisted case management during hospital discharge transition | 3 | | 746 | Significantly reduced HRR |
|  |  | 4 | | 1,072 | No differences in HRR. |
| Ponniah et al. 2007[95](5) | An enhanced medication management service for patients with heart failure | 4 | | * | (Significantly?) fewer unplanned admissions |
| Martínez et al. 2006 [96] (5) | Telemonitoring | 2 | | * | Significantly reduced HRR |
|  |  | 2 | | * | No differences in HRR. |
| Göhler et al. 2006[97](5) | Disease management | 6 | | 2,468 | Significantly reduced HRR |
|  |  | 1 | | 443 | Significantly increased HRR |
|  |  | 25 | | 4,657 | No significant differences in HRR. |
|  |  |  | |  | Overall: a statistically significant 8% decrease in all-cause HRR |
| Yu et al. 2006[98](5) | Disease management programs | 21 | | 4,445 | 11 trials: significantly reduced HRR by 29-85%; 9 trials: non-significantly reduced HRR by 2-36%; 2 trials: no reduction or increase in HRR |
| Gustafsson and Arnold 2004[99](3) | Heart failure clinics and outpatient management using nurse interventions With home visits No home visits | Total 18 | |  |  |
|  |  | 5 | | 844 | Significantly reduced HRR |
|  |  | 3 | | 374 | No differences in HRR. |
|  |  | 3 | | 653 | Significantly reduced HRR |
|  |  | 7 | | 1,553 | No differences in HRR. |
| Louis et al. 2003[100] (6) | Telemonitoring | 2 | | 201 | Fewer HRR /lower readmission charges. |
|  |  | 2 | | 427 | No difference in HRR. |
| Ferguson & Weinberger 1998[101](4) | Case management programs | 1 | | 282 | Significantly reduced HRR |
| **Coronary heart disease** | | | | | |
| Chiu & Newcomer 2007[94] (4) | Nurse-assisted case management during hospital discharge transition | 2 | | 486 | Significantly reduced HRR |
| Page et al. 2005[102](8) | Nurse-led cardiac clinics for patients with coronary heart disease. | 1 | | 597 | No diffrrences in HRR. |
|  |  | 1 | | 1173 | Reduced HRR |
| **Bronchial asthma** | | | | | |
| Smith et al. 2007[93](10) | Shared care between primary and specialty care in chronic disease management. | 1 | | 712 | No diffrences in HRR |
| Ferguson & Weinberger 1998[101](4) | Case management programs | 1 | | 104 | Significantly reduced HRR |
| **Chronic obstructive pulmonary disease** | | | | | |
| Hansen et al. 2011[43](8) | Predischarge patient education and discharge planning combined with follow-up phone calls, communication with care providers after discharge | 1 | | 149 | No sifferences in HRR. |
| Walters et al. 2010[103](11) | Guidelines for patients on how to recognise and self-manage exacerbations | 3 | | 394 | No significant diffrrences in HRR |
| Preyde et al. 2009[40](6) | Intensive community nurse-supported discharge planning for older patients with home visit | 1 | | * | No sifferences in HRR. |
| Chiu & Newcomer 2007[94](4) | Nurse-assisted case management during hospital discharge transition | 2 | | 334 | No sifferences in HRR. |
| Smith et al. 2007[93](10) | Shared care between primary and specialty care in chronic disease management. | 1 | | 135 | No diffrrences in all causeHRR: significantly reduced HRR for respiratory causes |
| Taylor et al. 2005[104] (9) | Nurse led disease management | 2 | | 313 | Significantly reduced HRR |
|  |  | 3 | | 323 | No differences in HRR |
| Smith et al. 2001[105](10) | Home care by outreach nursing | 1 | | * | No differences in HRR |
| **HIV** | | | | | |
| Young and Busgeeth 2010[106](9) | Home-based care | 2 | | 580 | No effect on hospitalizations or costs. |
|  |  | 1 | | 204 | Significantly reduced HRR |
| **Cancer** | | | | | |
| Smith et al. 2007[93](10) | Shared care between primary and specialty care in chronic disease management. | 1 | | 416 | Significantly reduced HRR in patients older than 70 years |
| **Diabetes** | | | | | |
| Smith et al. 2007[93](10) | Shared care between primary and specialty care in chronic disease management. | 1 | | 274 | No diffrrences in HRR |
| Griffin 1998[107](5) | Care in general practice vs hospital outpatient clinics | 2 | | 381 | 1 trial favored hospital care; |
|  |  |  | |  | 1 trial favored general practice care |
| **Stroke** | | | | | |
| Winkel et al. 2008[108](5) | Early discharge with home rehabilitation | 6 | | 689 | No significant effect on HRR |
| Larsen et al. 2006[109](2) | Early home-supported discharge | * | | * | No significant effect on HRR |
| **Unselected geriatric patients or patients with chronic disease** | | | | | |
| Smith et al. 2012[110,111](10) | Interventions for improving outcomes in patients with multimorbidity in primary care and community settings | | 1 | 543 | Significantly reduced HRR |
|  |  |  | 4 | 1,691 | No differences in HRR |
| Hansen et al. 2011[43](8) | Predischarge patient education reconciliation and discharge planning with phone follow-up | | 4 | 1,671 | Significantly reduced HRR. |
|  |  |  | 6 | 1,115 | No significant differences in HRR |
| Chisholm-Burns et al. 2010[111](8) | Pharmacists' effect on patient care | | 18 | * | Favorable results |
|  |  |  | 1 | * | Not favorable results |
|  |  |  | 1 | * | Mixed results |
|  |  |  | 15 | * | No effect on HRR |
| Batty 2010[112](6) | Geriatric evaluation and management in clinic Home based primary care model Clinic and home visits by a multi-disciplinary team Patients seen in hospital and clinic and/or home | | 1 | 98 | No effect on HRR |
|  |  |  | 1 | 1,966 | No effect on HRR |
|  |  |  | 1 | 543 | Significantly reduced HRR |
|  |  |  | 1 | 941 | No effect on HRR. |
|  |  |  | 2 | 1,489 | Significantly reduced HRR |
|  |  |  | 1 | 166 | No effect on HRR. |
| Oeseburg et al. 2009[113](6) | Cordination of care of impaired older people or chronic patients in the community by education, self-management, home visits or telephone contact | | 1 | 199 | Small significant reduction in HRR |
|  |  |  | 5 | 14,438 | No effect on HRR |
| Preyde et al. 2009[40](6) | Discharge planning and home follow-up | | 3 | * | Significantly reduced HRR. |
|  | Geriatric assessment with home intervention | | 2 |  | No significant differences in HRR |
|  | Discharge planning, home telephone follow-up | | 1 |  | No significant differences in HRR |
| Hsiao and Boult 2008[114](2) | Effects of quality of care on outcomes in primary care | | 1 | 776 | Significantly reduced emergent hospital admissions |
| Garcıa-Lizana and Sarrıa-Santamera 2007[92](6) | Use of information / communication technologies (internet, videoconference, interactive media, telemedicine). The patients are receptors of the activity or the source of data. | | 1 | 104 | Significantly reduced HRR. |
|  |  |  | 1 | 212 | No significant differences in HRR |
| Chiu & Newcomer 2007[94](3) | Nurse-assisted case management during hospital discharge transition in unselected patients | | 3 | 2,506 | Significantly reduced HRR. |
|  |  |  | 2 | 961 | No difference in HRR |
| Worrall and Knight 2006[115](8) | Continuity of care for older patients in family practice | | 2 | 1.071 | Significantly reduced emergency HRR |
| Hastings et al. 2005 [116] (6) | Emergency department-based discharge planning or comprehensive geriatric assessment or nurse case management AND home based follow up | | 1 | * | Significantly reduced HRR. |
|  |  |  | 3 | * | No difference in HRR |
| Richards & Coast 2003[41](5) | Outpatient geriatric evaluation / management | | 2 | 320 | No differences in HRR. |
|  |  |  | 1 | 324 | Shorter length of stay in hospitals (p<0.05) |
| Berendsen et al. 2002[117](5) | Hospital at home | | 4 | 501 | 3 trials: no significant difference in HRR. |
|  |  | |  |  | 1 trial: fewer HRR |
| Parker et al. 2000[37](6) | Hospital at home for either early discharge from acute care hospitals, or admission avoidance. Rehabilitation Day hospital | | 4 | * | Significantly reduced HRR. |
|  |  |  | 5 | * | No differences in HRR |
|  |  |  | 1 | * | No differences in HRR |
|  |  |  | 4 | * | No differences in HRR |
| Scott 1999[42](4) | Community/home based geriatric evaluation and management utpatient / community based geriatric consultations | | 3 | 1,036 | No differences in HRR at 3–12 months |
|  |  |  | 4 | 1,300 | No differences in HRR at 2–36 months. |
|  |  |  | 1 | 762 | Significantly reduced HRR at 6 months |
| Ferguson & Weinberger 1998[101](4) | Case management programs in primary care of patients with chronic disease | | 1 | 1,396 | Significantly *increased* HRR |
|  |  |  | 4 | 2,248 | No significant effect on HRR |
| Hughes et al. 1997[118](6) | Home care | | 4 | 256 | Significant reduction in hospital days. |
|  |  |  | 7 | 3,179 | Non-significant reduction |

HRR – Hospital readmission rates. RCT- Randomized controlled trials * - not given
